# Anaerobic peroxisomes in *Entamoeba histolytica* metabolize *myo*-inositol

**DOI:** 10.1371/journal.ppat.1010041

**Published:** 2021-11-15

**Authors:** Zdeněk Verner, Vojtěch Žárský, Tien Le, Ravi Kumar Narayanasamy, Petr Rada, Daniel Rozbeský, Abhijith Makki, Darja Belišová, Ivan Hrdý, Marie Vancová, Corinna Lender, Constantin König, Iris Bruchhaus, Jan Tachezy

**Affiliations:** 1 Department of Parasitology, Faculty of Science, Charles University, BIOCEV, Vestec, Czech Republic; 2 Department of Cell Biology, Faculty of Science, Charles University, BIOCEV, Vestec, Czech Republic; 3 Biology Centre, Czech Academy of Sciences, Institute of Parasitology, Ceske Budejovice, Czech Republic; 4 Bernhard Nocht Institute for Tropical Medicine, Hamburg, Germany; The University of Tokyo, JAPAN

## Abstract

*Entamoeba histolytica* is believed to be devoid of peroxisomes, like most anaerobic protists. In this work, we provided the first evidence that peroxisomes are present in *E*. *histolytica*, although only seven proteins responsible for peroxisome biogenesis (peroxins) were identified (Pex1, Pex6, Pex5, Pex11, Pex14, Pex16, and Pex19). Targeting matrix proteins to peroxisomes is reduced to the PTS1-dependent pathway mediated via the soluble Pex5 receptor, while the PTS2 receptor Pex7 is absent. Immunofluorescence microscopy showed that peroxisomal markers (Pex5, Pex14, Pex16, Pex19) are present in vesicles distinct from mitosomes, the endoplasmic reticulum, and the endosome/phagosome system, except Pex11, which has dual localization in peroxisomes and mitosomes. Immunoelectron microscopy revealed that Pex14 localized to vesicles of approximately 90–100 nm in diameter. Proteomic analyses of affinity-purified peroxisomes and *in silico* PTS1 predictions provided datasets of 655 and 56 peroxisomal candidates, respectively; however, only six proteins were shared by both datasets, including *myo*-inositol dehydrogenase (*myo*-IDH). Peroxisomal NAD-dependent *myo*-IDH appeared to be a dimeric enzyme with high affinity to *myo*-inositol (Km 0.044 mM) and can utilize also *scyllo*-inositol, D-glucose and D-xylose as substrates. Phylogenetic analyses revealed that orthologs of *myo*-IDH with PTS1 are present in *E*. *dispar*, *E*. *nutalli* and *E*. *moshkovskii* but not in *E*. *invadens*, and form a monophyletic clade of mostly peroxisomal orthologs with free-living *Mastigamoeba balamuthi* and *Pelomyxa schiedti*. The presence of peroxisomes in *E*. *histolytica* and other archamoebae breaks the paradigm of peroxisome absence in anaerobes and provides a new potential target for the development of antiparasitic drugs.

## Author summary

*E*. *histolytica* colonizes the human large intestine upon ingestion of amebic cysts. Trophozoites dwell in the hypoxic *milieu* of the intestinal lumen and can invade the epithelium to cause potentially lethal amebiasis. As an anaerobe, *E*. *histolytica* is dependent on glycolysis to generate ATP, whereas mitochondria are present in a greatly reduced form called mitosomes. Peroxisomes are believed to be absent in *E*. *histolytica*, as in other anaerobes, supported by the lack of typical peroxisomal pathways, including β-oxidation and catalase. However, we identified seven proteins involved in peroxisome biogenesis, the peroxins, and our investigations of their cellular localization indicated that *E*. *histolytic*a possesses the anaerobic form of peroxisomes that we recently discovered in free-living anaerobic relatives. Proteomic analysis of anaerobic peroxisomes revealed the presence of highly active homodimeric *myo*-inositol dehydrogenase (*myo*-IDH), which catalyzes NAD-dependent oxidation of *myo*-inositol and other cyclitols. Since upstream and downstream pathways are absent in anaerobic peroxisomes, the role of *myo*-IDH is most likely to maintain intraperoxisomal redox potential. Discovery of anaerobic peroxisomes in *E*. *histolytica* and identification of *myo*-IDH that is absent in host cells provide a new avenue for the development of strategies against the parasite.

## Introduction

*Entamoeba histolytica* is a causative agent of amoebiasis, one of the most prevalent parasitic diseases of humans. Over 65,000 lethal cases of amoebiasis per year have been reported worldwide [1,2], and *E*. *histolytica* prevalence has been estimated to reach 3.55% globally [3]. *E*. *histolytica* colonizes the human large intestine, and upon a not-well-understood trigger, the trophozoites invade the mucous barrier and cause dysentery and eventually extraintestinal amoebiasis [4]. Due to its adaptation to oxygen-poor environments and its parasitic lifestyle, the cellular organelles and metabolism of *E*. *histolytica* are highly modified [5]. *Entamoeba* does not possess classical mitochondria; instead, the cells harbor minimal versions, called mitosomes. The organelles do not contain DNA or exhibit classical energy metabolism, yet they are surrounded by a double membrane [6–8]. The only known mitosomal function is sulfate activation [9,10]. Energy metabolism is based on glycolysis in the cytosol, where glucose is converted to pyruvate, and extended glycolysis, that includes conversion of pyruvate to acetyl-CoA by pyruvate:ferredoxin oxidoreductase; acetyl-CoA is then utilized for ATP production by acetyl-CoA synthase [11]. The *E*. *histolytica* cytosol contains numerous vesicles, lysosomes, endosomes, and multivesicular bodies, whereas the endoplasmic reticulum (ER) and Golgi apparatus (GA) are difficult to recognize and, in the case of the Golgi, were once thought to be absent [12]. However, later studies showed that the ER forms a tubular structure similar to the ER of other eukaryotic cells [13,14], and a GA was visualized in the form of separated vesicles bearing some GA markers, such as Golgi-associated coatomer protein ɛ-COP [15,16]. Peroxisomes are single membrane-bound multifunctional organelles. A defining feature of classical peroxisome is the presence of peroxide-generating and detoxifying pathways with the marker enzyme catalase [17,18]. However, some specialized forms of the organelle do not contain these key pathways and play various different roles: glycosomes of trypanosomatids are known to contain the first six or seven glycolytic enzymes [19], and plant glyoxysomes engage in the glyoxalate cycle, whereas Woronin bodies of filamentous fungi serve as a physical barrier between two cells upon hyphal wounding [20].

Despite the wide range of metabolic diversity, all peroxisomes share a common *de novo* biogenesis pathway mediated by a set of specific proteins named peroxins (PEXs). In the model organism *Saccharomyces cerevisiae*, peroxisomal matrix proteins are synthesized in the cytosol and recognized by the receptor proteins Pex5 and Pex7 with C-terminal peroxisomal targeting signal (PTS)1 and N-terminal PTS2, respectively [21]. The receptor-cargo is then recruited by docking proteins (Pex13, Pex14 and Pex17) and a transient pore composed of Pex5-Pex14 is formed. The cargo is released into the lumen of peroxisomes while RING finger proteins (ubiquitin ligases Pex2, Pex10 and Pex12) are assembled onto the transition pore. The receptor is recycled from the membrane upon monoubiquinylation of a conserved cysteine or degraded upon polyubiquitinylation of a conserved lysine. The process of ubiquitinylation involves RING proteins, and ubiquitin-conjugated Pex4 is anchored to the membrane via Pex22. Release of the receptor from the membrane is facilitated by the Pex1/6 heterohexamer anchored to the membrane via Pex15, which is also responsible for deubiquitinylation [21]. Membrane proteins are incorporated into the peroxisomal membrane by the cycling chaperone Pex19, which recognizes the membrane PTS and recruits the protein to a membrane-anchored Pex3 [18]. The trafficking of glycosylated proteins from the ER is mediated by Pex16, which is absent in *Saccharomyces* species [18,22]. Similar to mitochondria, peroxisomes can undergo fission, exploiting dynamin-like proteins that are recruited by Pex11 [18].

Peroxisomes have been widely reported from aerobic organisms, and their evolution was suggested to be tightly connected with oxygen-dependent metabolic pathways, particularly β-oxidation of fatty acids, redox homeostasis, and bioenergetics [23,24]. Moreover, it has been shown in human mutant fibroblasts that peroxisomes could be formed by a fusion of vesicles derived from both the ER and mitochondria [25]. In this case, Pex3 and Pex14 were first inserted into the mitochondrial outer membrane, which later buds in the form of a vesicle; moreover, Pex16-containing vesicles were shown to originate from the ER. Mature peroxisomes were determined to be a union of the two distinct classes of pre-peroxisomal vesicles [25].

Peroxisomes are generally thought to be absent in anaerobic organisms, including *E*. *histolytica* [26]. Recently, however, anaerobic peroxisomes were described in the free-living anaerobic amoeba *Mastigamoeba balamuthi*, which bear an anaerobic form of mitochondria named hydrogenosomes [27]. *M*. *balamuthi* peroxisomes contain a major part of the pyrimidine biosynthesis pathway, enzymes required for coenzyme A synthesis, and carbohydrate metabolism, while β-oxidation and catalase are absent [27]. Moreover, the presence of peroxisomes was noted in another anaerobic member of the Archamoebae, *Pelomyxa schiedti* [28]. The discovery of anaerobic peroxisomes in free-living archamoebae prompted us to search for peroxisomes in evolutionarily related parasitic *Entamoeba* species. Indeed, we identified a minimal set of *pex* genes that provide *E*. *histolytica* with the capacity to form peroxisomes, and we partially characterized the peroxisomal proteome. The common feature of anaerobic peroxisomes in Archamoebae appears to be the metabolism of *myo*-inositol.

## Results

### Identification of putative PEX proteins in the genome of *Entamoeba* species

**Fig 1:**
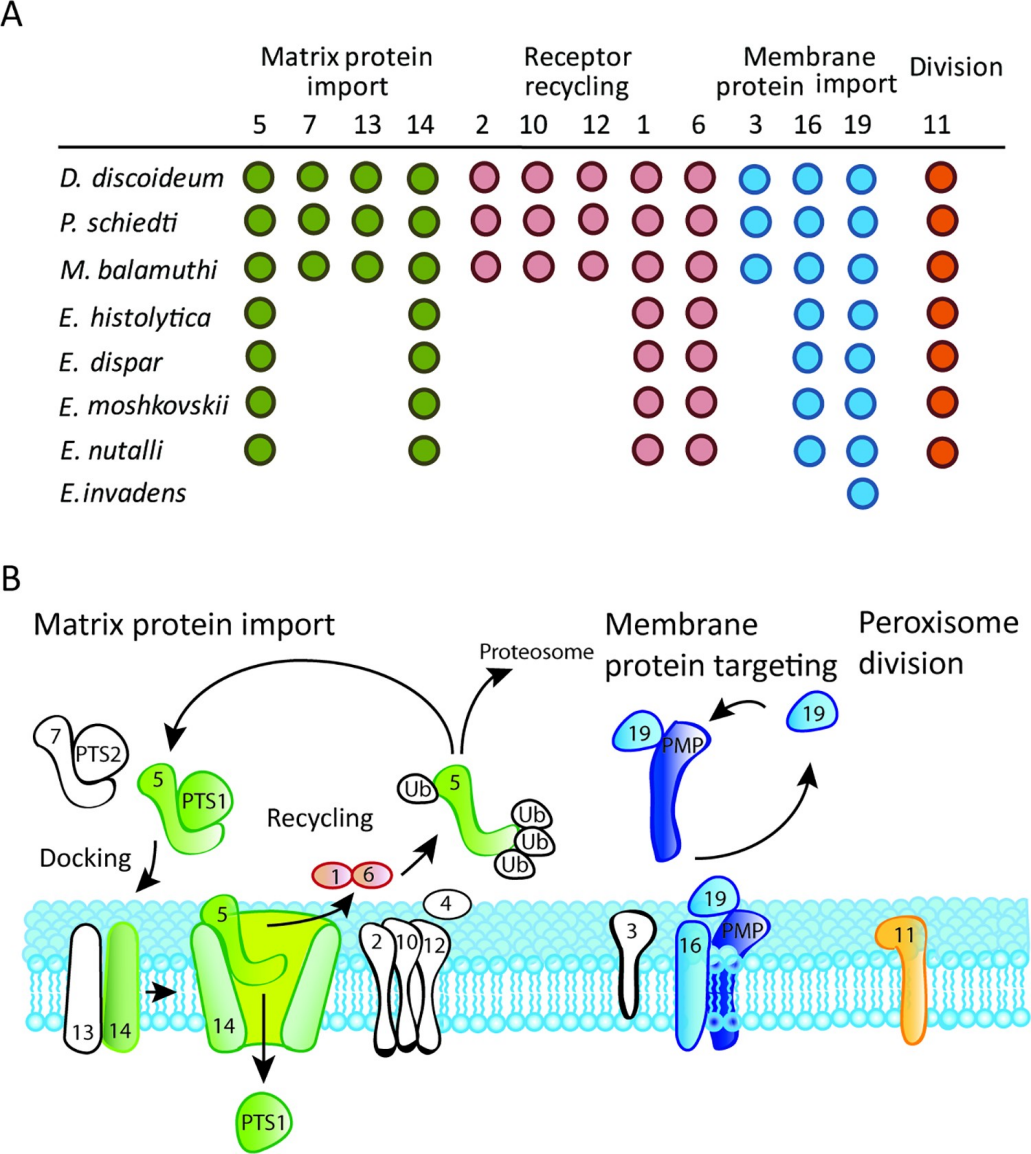
Prediction of *PEX* coding genes. A. Predicted *PEXs* in *E*. *histolytica* and related Evosea species. B. Scheme of the peroxisomal machinery identified in *Entamoeba* species except for *E*. *invadens*. The white color indicates PEXs that were not identified. Ub, ubiquitin; PMP, peroxisomal membrane protein.

**Fig 2:**
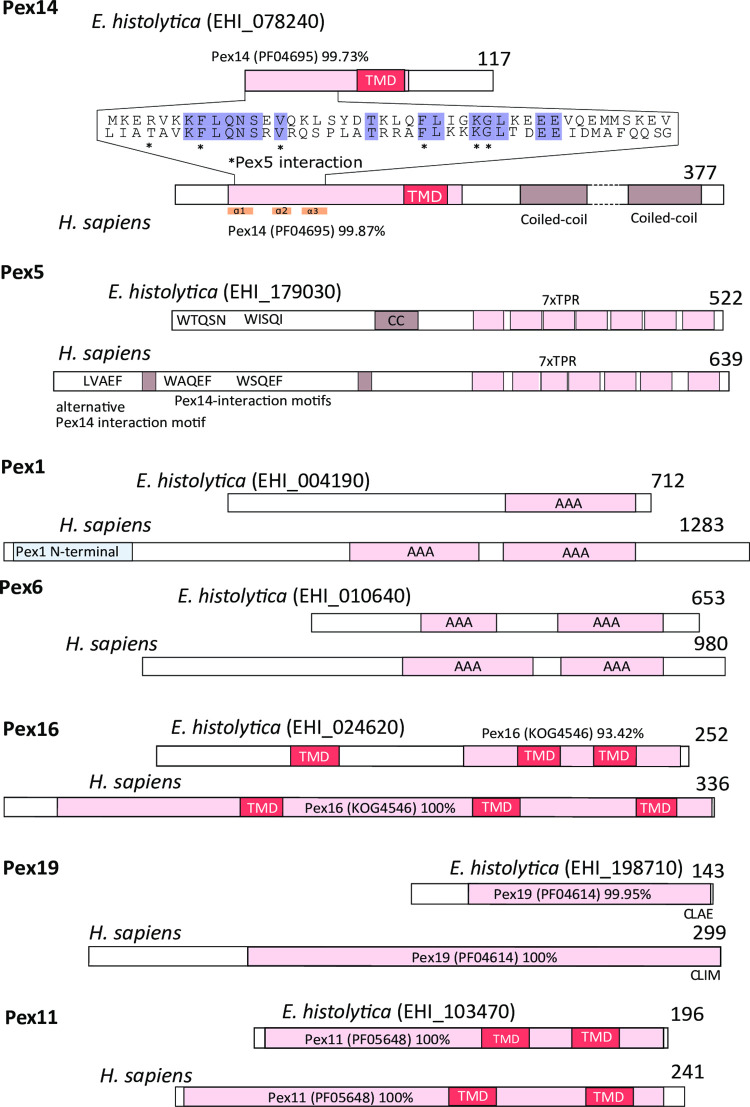
Domain structures of *E*. *histolytica* and *Homo sapiens* PEXs. PFAM domain identifiers (in brackets) and probability percentages are indicated. TMD, transmembrane domain; TPR, tetratricopeptide domain; stars indicate Pex14 amino acid residues that are involved in Pex5 interactions [30].

**Fig 3:**
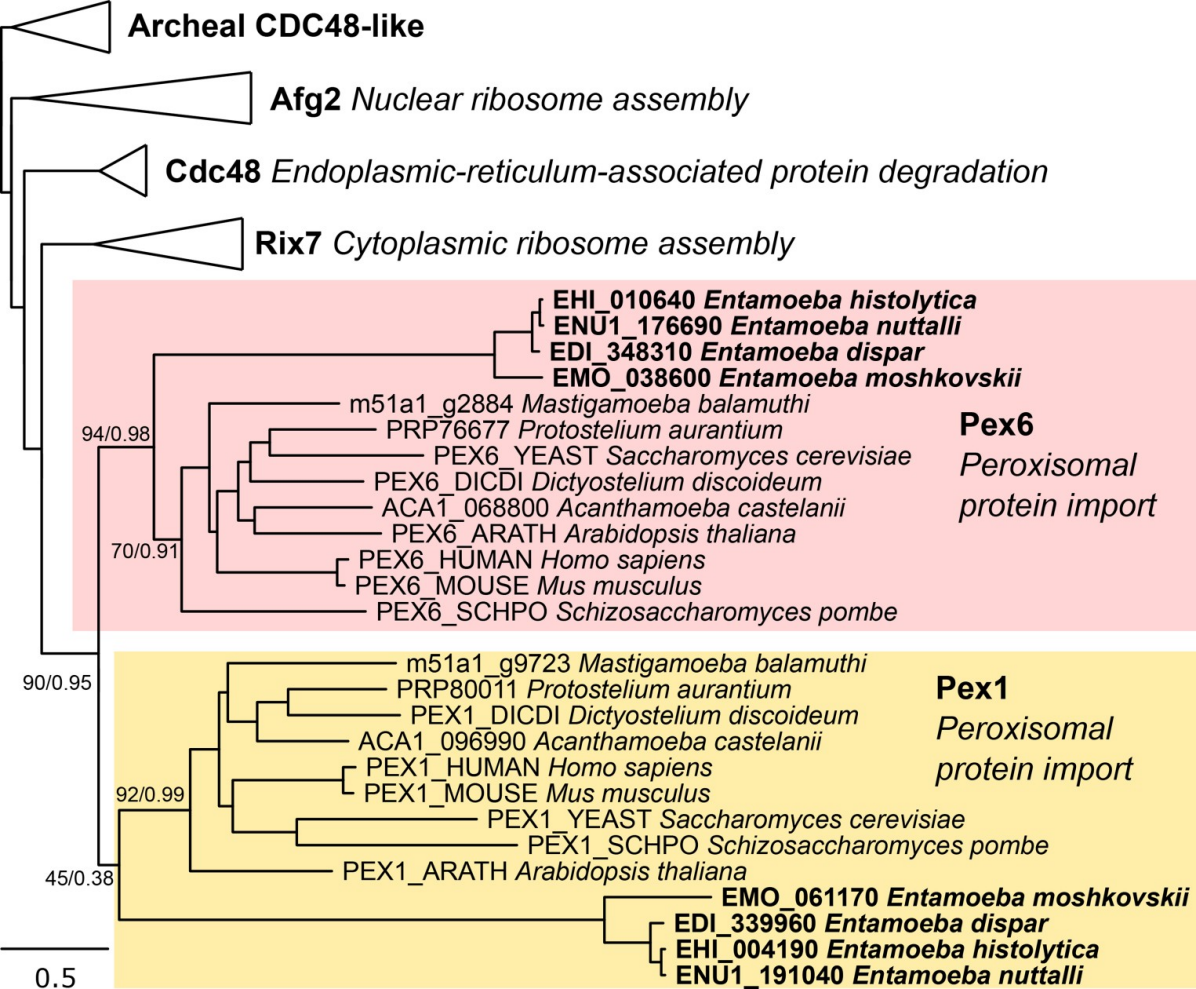
Phylogeny of Pex1, Pex6 and other proteins with AAA domains. The maximum likelihood tree was inferred with IQ-TREE using 51 protein sequences and 522 positions. Numbers at nodes of the tree indicate statistical support in the form of an ultrafast bootstrap of the IQ-Tree and posterior probability of the PhyloBayes analysis.

Initially, we searched for PEXs in the genomes of *E*. *histolytica* and four other *Entamoeba* species, the human parasites *E*. *dispar* and *E*. *moshkovskii*, a parasite of non-human primates *E*. *nuttalli*, and a reptile pathogen *E*. *invadens*. The searches, which were performed using PEXs of a related amoebozoan *M*. *balamuthi* and HMMs of other eukaryotic PEX proteins as queries, revealed the presence of a reduced set of seven PEXs in all primate species (Fig 1A and S1 Table). The identified PEXs include members of each functional category (Fig 1B). Components of matrix protein imports were represented by Pex5 and Pex14. Pex5 is a soluble receptor that recognizes the PTS1 of proteins to be imported by binding to a domain formed by tetratricopeptide repeats within its C-terminal half (Fig 2). A transient pore constituting of Pex14 and Pex5 is formed after the interaction of Pex5 with the docking complex by binding to characteristic motifs in the N-terminal half of Pex14 (Fig 2) [29]. Members of membrane protein import include Pex16, a protein involved in embedding of peroxisomal membrane proteins, and Pex19, a membrane protein receptor. Although *E*. *histolytica* Pex19 is considerably shorter than the human ortholog, its classification to the Pex19 family (PF04614) is strongly supported, although it lacks the N-terminal domain that is required for interaction with Pex3 [30,31] (Fig 2). We did not identify any member of the RING finger proteins of the receptor recycling machinery (Pex2, Pex10, Pex12); however, we detected putative Pex1 and Pex6, which are responsible for receptor deubiquitinylation (Fig 1B). Because Pex1 and Pex6 possess AAA domains that are present in other proteins and may lead to false-positive identification, we also performed a phylogenetic analysis of Pex1/Pex6 orthologs and related AAA proteins with different functions (Fig 3). This analysis supported the correct identification of putative Entamoeba Pex1 and Pex6. Finally, we identified Pex11, a multi-purpose protein, which is involved in peroxisomal proliferation by elongation and fission and metabolism via the formation of a membrane channel [32,33]. Surprisingly, we did not identify any PEXs in *E*. *invadens* except for Pex19 (Fig 1A). All identified proteins showed apparent divergence from canonical sequences of yeast and human orthologues, with protein sequence identity ranging from 8–23% (S2 Table). Taken together, our searches suggested that *E*. *histolytica* and three other *Entamoeba* species have a minimal set of seven PEXs that might be able to facilitate peroxisomal biogenesis, while *E*. *invadens* most likely lacks these organelles, with only Pex19 being present.

### Identified PEXs are all expressed

Identification of the putative PEX proteins prompted us to test whether the proteins are expressed and to what extent. Total RNA was isolated using cells upon reaching a monolayer in the culture (approximately 48 hours). RT-qPCR showed that under these conditions, all *PEXs* were expressed, but the relative expression level was lower than that of the selected housekeeping enzymes pyruvate kinase, phosphofructokinase, and alcohol dehydrogenase (S3 Table). The low expression level of putative *PEXs* corresponded to previously published transcriptomic data [34]. The highest expression was observed for *Pex11*, while *Pex19* was the least expressed. Because the regulation of genes involved in the same function might be linked, we decided to clone *Pex5*, *11*, *14*, *16*, and *19* into a vector that allowed their overexpression with a polyHis-tag under the control of a strong lectin promotor [34,35]. The transfectants revealed a relative *PEX* overexpression from 31 (*Pex11*) to 580 (*Pex5*)-fold (S4 Table). However, overexpression of a particular *PEX* gene had a negligible effect on native expression of other *PEXs*, with the exception of *Pex14*, overexpression of which was accompanied by slightly increased expression of *Pex16* (3-fold).

### PEX proteins in *E*. *histolytica* localized to vesicles distinct from mitosomes, the ER and endosomal/lysosomal structures

**Fig 4:**
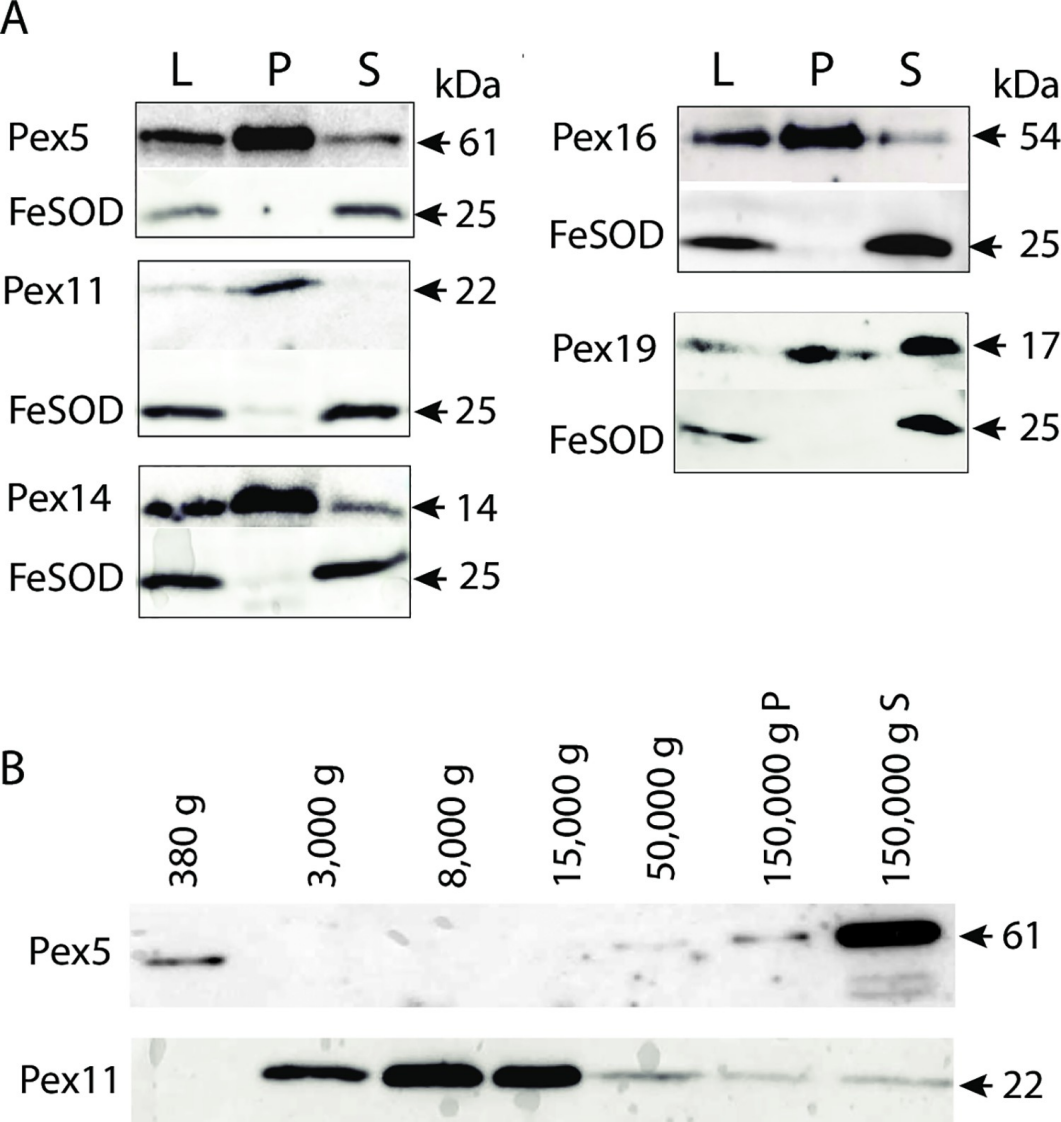
Expression of tagged PEXs in *E*. *histolytica*. A. Western blot analysis of cell lysate (L), organelle (P) and cytosolic soluble (S) fractions. Fe-superoxide dismutase (FeSOD) was used as the cytosolic marker. B. Western blot analysis of Pex5 and Pex11 in seven fractions isolated by differential centrifugation using 0.05% Tween-20 to limit protein aggregation.

The subcellular localization of PEXs was studied in transfectants expressing Pex5, 11, 14, 16, and 19. First, we analyzed recombinant proteins using immunoblot analysis of the sedimentable (organelle) and soluble (cytosolic) fractions of transfectants (Figs 4 and S1). This analysis confirmed that all PEXs are expressed on the protein level, and more importantly, Pex5, 11, 14, and 16 were predominantly present in the sedimentable fraction, whereas the signal for Pex19 was comparably strong in the organellar fraction and the cytosol. Iron-containing superoxide dismutase (Fe-SOD) was used as a cytosolic marker (Fig 4A). For the soluble receptor Pex5, we expected to observe higher amounts of this protein in the cytosol. Thus, we performed a more detailed cell fractionation using differential centrifugation in five consecutive steps at 380–150,000 x g, and we used 0.05% Tween-20 in the buffer to limit protein aggregation. Under these conditions, a majority of Pex5 was in the soluble fraction, while the majority of the peroxisomal membrane Pex11 was present in sedimentable fractions (Fig 4B). This result indicates that Pex5 is transiently associated with organelles.

**Fig 5:**
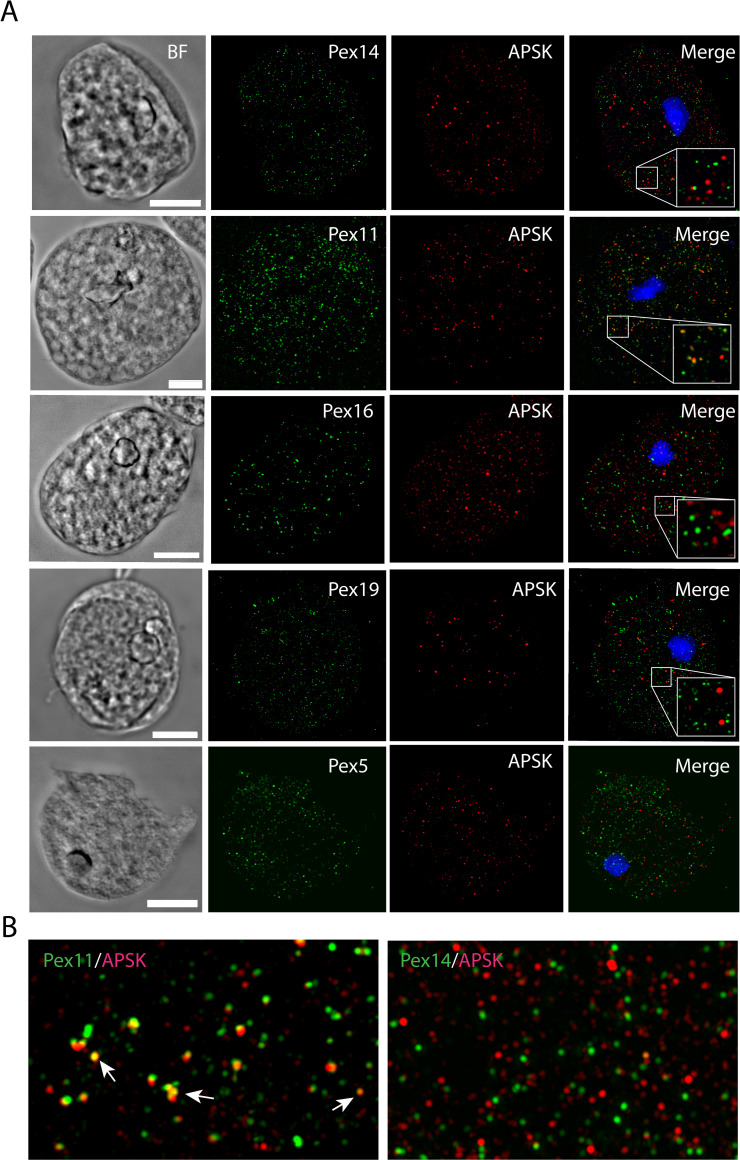
Cellular localization of PEXs in *E*. *histolytica*. A. Immunofluorescence microscopy of His-tagged PEXs and the mitosomal marker APSK. B. Structured illumination microscopy of Pex11, Pex14, and APSK. BF, bright field. Scale bar: 10 μm.

**Fig 6:**
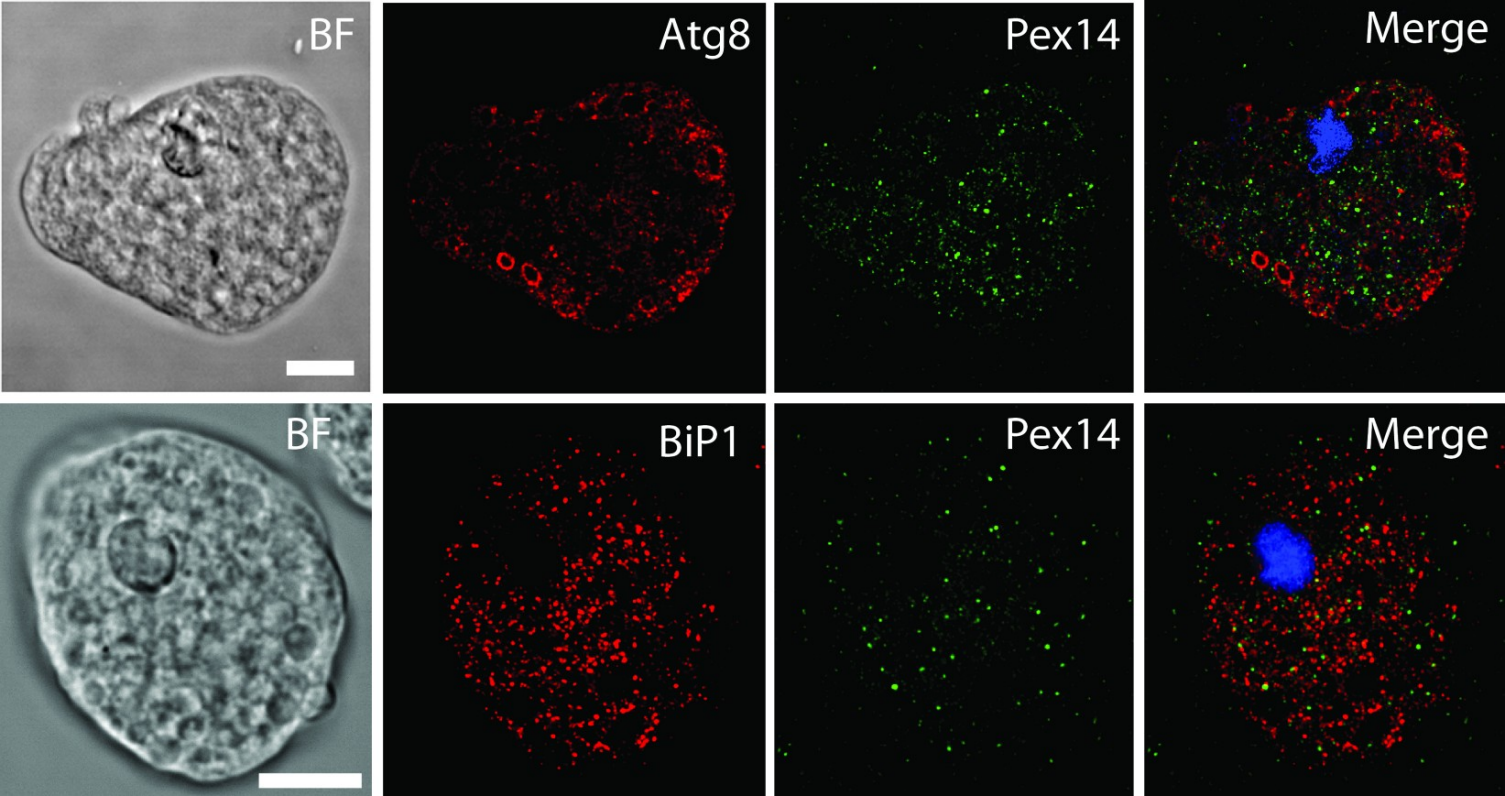
Confocal microscopy of vesicles labeled with Pex14, ER marker BiP1 and lysosomal marker Atg8. BF, bright field. Scale bar: 10 μm.

**Fig 7:**
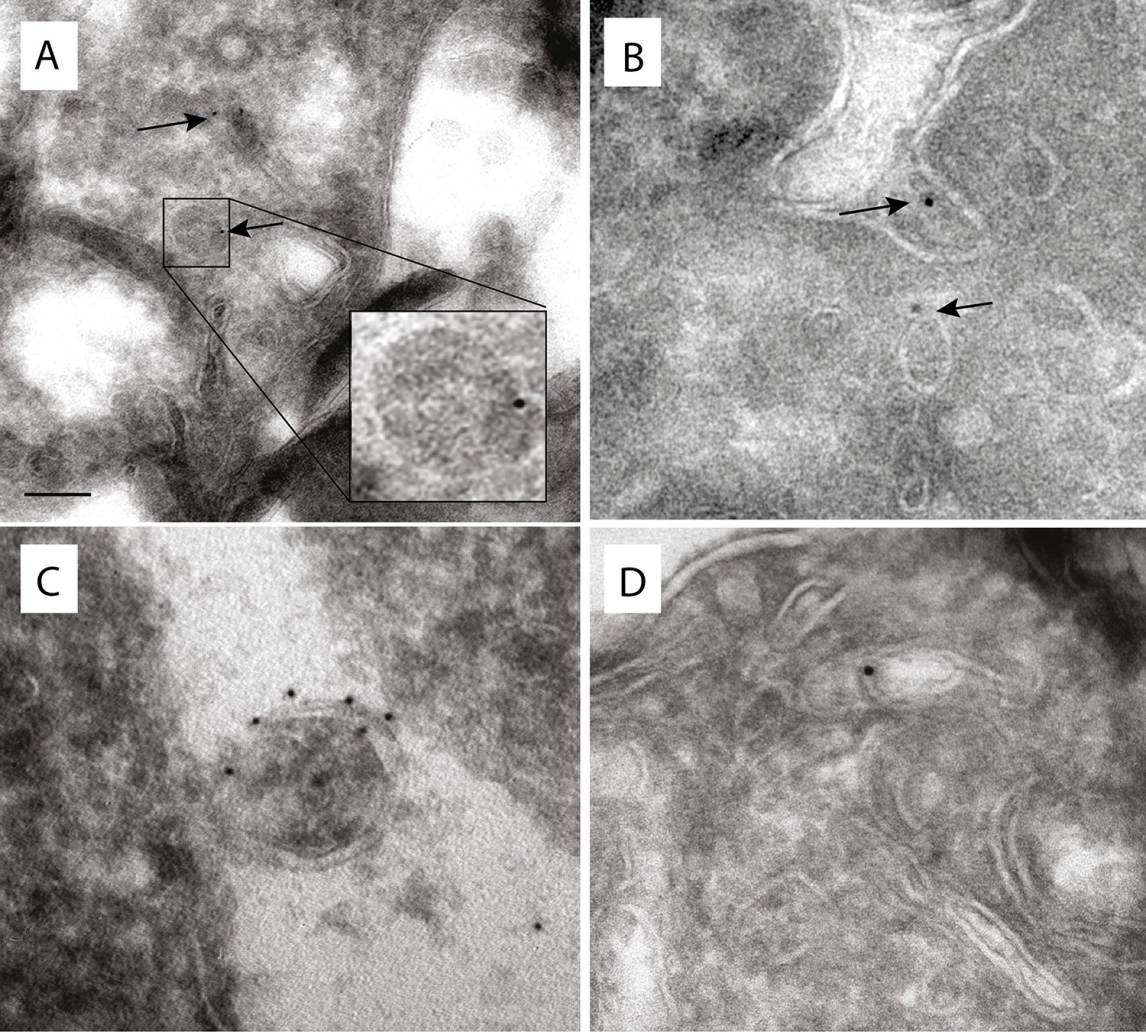
Immunoelectron microscopy detection of Pex14 and Pex16 in *E*. *histolytica*. Pex14 (A,B) was detected using Ni-NTA conjugated with 5 nm gold particles (A) or anti-V5 antibody (B). Pex16 (C,D) was detected using rabbit (C) and mouse (B) anti-His antibodies and the corresponding antibody conjugated with 5 nm gold particles. Scale bar: 100 nm.

More detailed localization of PEXs was investigated using confocal microscopy. First, we attempted to distinguish putative peroxisomes from mitosomes (Fig 5A). We observed that both PEXs and APSK, a marker protein of mitosomes, labeled small round vesicles of similar sizes, but none of the PEXs colocalized with APSK. The Pearson’s correlation coefficient in colocalized volume (PCC) ranged between -0.072 to 0.023, which indicates no or negligible correlation between PEXs and APSK signals (S2 and S5 Figs and S5 Table). The only exception was Pex11, which partially localized to mitosomes with weak PCC *r* = 0.241. This observation was further supported by structured illumination microscopy (SIM)(Fig 5B). While Pex14 did not colocalize with the APSK signal, Pex11 was partially associated with mitosomes (Fig 5B). Pex14 also did not colocalize with other cellular vesicles and vacuoles labeled with Atg8(PCC *r* = 0.033) [36] and the ER marker BiP1(PCC *r* = 0.021) [37] (Figs 6 and S2 and S5 Tables). The number of putative peroxisomes counted for Pex14-labeled organelles was approximately 267±60 per 100 μm^2^ (mean ± S.D., n = 25). The specific vesicular localization of Pex14 and Pex16 was further supported by immunoelectron microscopy. Weak but highly specific signals for His-tagged Pex14 were associated with the membranes of round vesicles of approximately 90–100 nm in diameter (Fig 7A and 7B). Similarly, His-tagged Pex16 was observed at the vesicular membrane. We also observed Pex16-labeled vesicles that were partially surrounded by two more membranes, with Pex16 signals on the proximal and distal membranes. The character of these structures is unclear though they remind lamellar derivatives of the endoplasmic reticulum that deliver Pex16 and other membrane proteins for *de novo* formation of putative peroxisomes (Fig 7C and 7D) [38].

### Proteomic analysis of the peroxisomal fraction and PTS1 predictions revealed the presence of inositol dehydrogenase in the peroxisomal matrix

**Fig 8:**
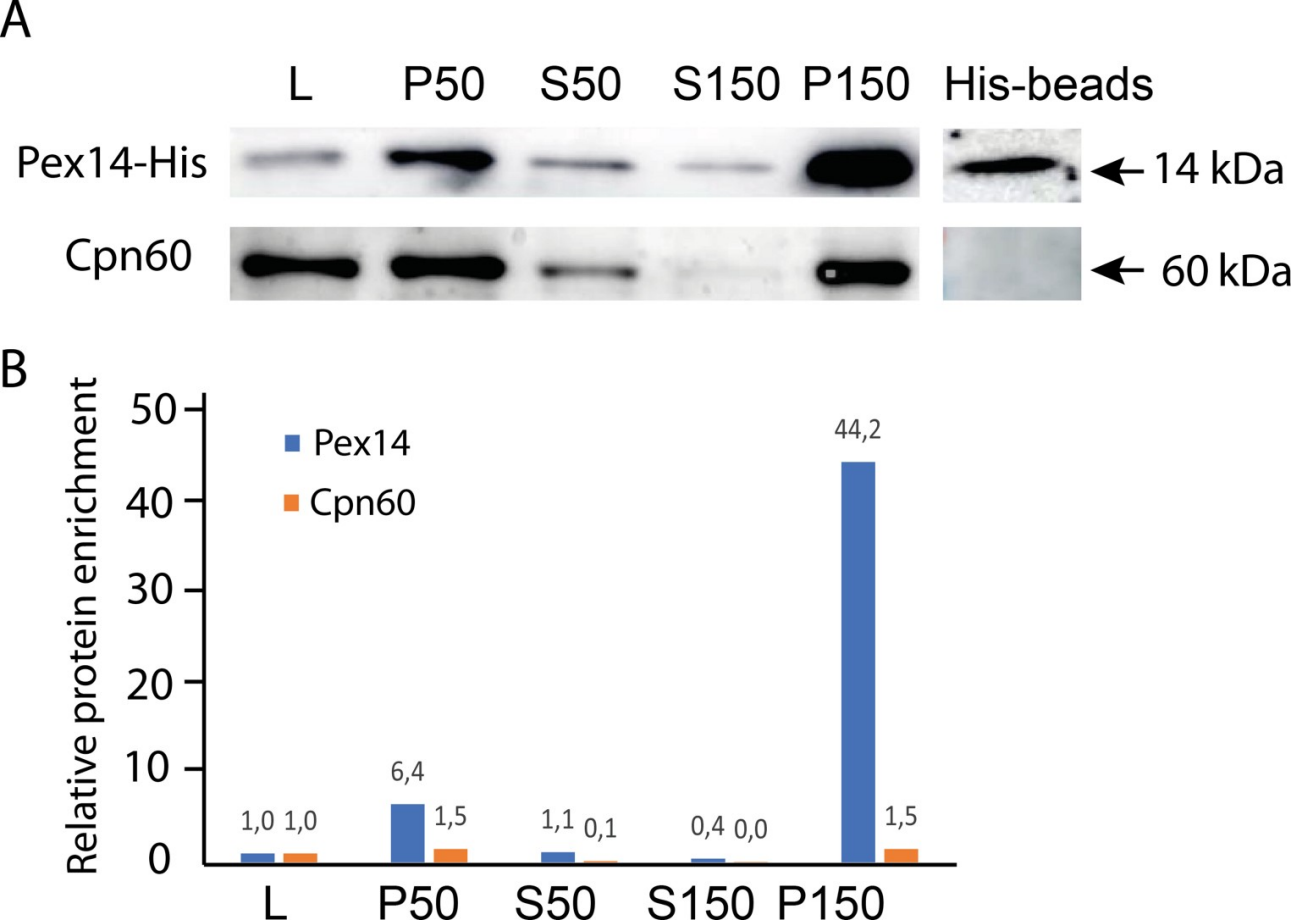
Affinity purification of peroxisomes. A. Lysate of cells (L) expressing His-tagged Pex14 was used for differential centrifugation at 50 000 and 150 000 x g, and pellet (P) and soluble (S) fractions were analyzed by western blotting. In the last step, peroxisomes were purified using anti-His antibody-conjugated beads (His-beads) that were used for mass spectrometry. Antibodies against Cpn60 were used as mitosomal markers. B. Relative protein enrichment was calculated with densitometry of Pex14-His and Cpn60 signals.

Initial experiments to isolate putative peroxisomes based on differential and gradient centrifugation did not allow effective separation of Pex14-localized organelles and mitosomes. Therefore, we decided to use affinity purification of the organelles. Pex14 was expressed with a C-terminal poly-His or V5 tag facing the cytosol [39], and putative peroxisomes were isolated from cell homogenates using magnetic beads conjugated with the corresponding antibody. In a pilot experiment, all steps were monitored by western blotting to assess enrichment of putative peroxisomes using an anti-His antibody and mitosome contamination using an anti-Cpn60 antibody (Figs 8 and S1B). Western blot analysis showed approximately 40-fold enrichment of the Pex14-His signal after differential centrifugation (150,000 x g pellet, Fig 8B), and the Pex14-His signal was separated from Cpn60 using anti-His antibody-conjugated beads (Fig 8A). A summary of LFQ mass spectrometry analyses of putative peroxisome-enriched fractions with His-tagged Pex14 (seven experiments) and V5-tagged Pex14 (six experiments) is given in S6 Table. In two experiments with V5-tagged Pex14, we omitted Tween 20 to estimate, which proteins are loosely associated with the organelle surface. Altogether, 655 proteins were identified in the peroxisome-enriched fraction, of which 24 were observed only in experiments without Tween 20 (S6 Table). In addition to tagged Pex14, the dataset contained two other PEXs, Pex5 and Pex11. Interestingly, although orthologs of RING complex E3 ubiquitin ligases Pex2, Pex10 and Pex12 and E2 ubiquitin-conjugating protein Pex4 are absent in *E*. *histolytica*, a single putative E3 ubiquitin ligase EHI_030770 and two ubiquitin-conjugating enzyme family proteins, EHI_083560 and EHI_048700, were identified in the proteome.

Comparison with the proteome of *M*. *balamuthi* peroxisomes [27] revealed eight proteins in common, such as putative inositol dehydrogenase (IDH), long-chain fatty acid-CoA ligase, and malate dehydrogenase (S6 Table). We were also interested in the overlap between our dataset and the previously reported mitosomal proteome [9]. This comparison revealed 35 common proteins, of which four proteins detected in the mitosome-enriched fraction were between our top 100 peroxisomal candidates with the highest cumulative score (S6 Table). These proteins included putative IDH, which was previously localized in the cytosol [9], a hypothetical protein EHI_103470, which we recognized as Pex11, a hypothetical protein (EHI_170120) that was reported as the mitosomal membrane protein [40], and a microtubule-binding protein (S6 Table).

Next, we performed *in silico* prediction of PTS1 signals in proteins predicted in all available *Entamoeba* species using a machine learning algorithm (PTS1 ML) optimized according to the *M*. *balamuthi* peroxisomal proteome [27]. We rationalized that a high score for PTS1 ML prediction in *E*. *invadens* that seems to lack peroxisomes likely represents a false positive signal and that the absence of proteins in *E*. *invadens* in comparison to other species might be related to the absence of peroxisomes. Thus, the PTS1 ML score for *E*. *invadens* proteins was subtracted from the corresponding PTS1 ML score for *E*. *histolytica* (S7 Table). In addition, we used three other available tools for PTS1 prediction [41,42]. These analyses provided a set of 56 proteins with putative PTS1 predicted by at least two tools (S7 Table). The comparison of this dataset and proteomic data (S6 Table) revealed only six proteins present in the proteome of the putative peroxisome-enriched fraction with the predicted PTS1 signal using our strict criteria. These proteins include five hypothetical proteins of unknown function (EHI_045060, EHI_050510, EHI_183900, EHI_185440, and EHI_161040) and putative IDH (EHI_125740). The absence of genes for IDH and the hypothetical protein EHI_045060 in the *E*. *invadens* genome further support its possible associations with putative peroxisomes of *E*. *histolytica* (S7 Table).

### Import of IDH to putative peroxisomes is PTS1-dependent

**Fig 9:**
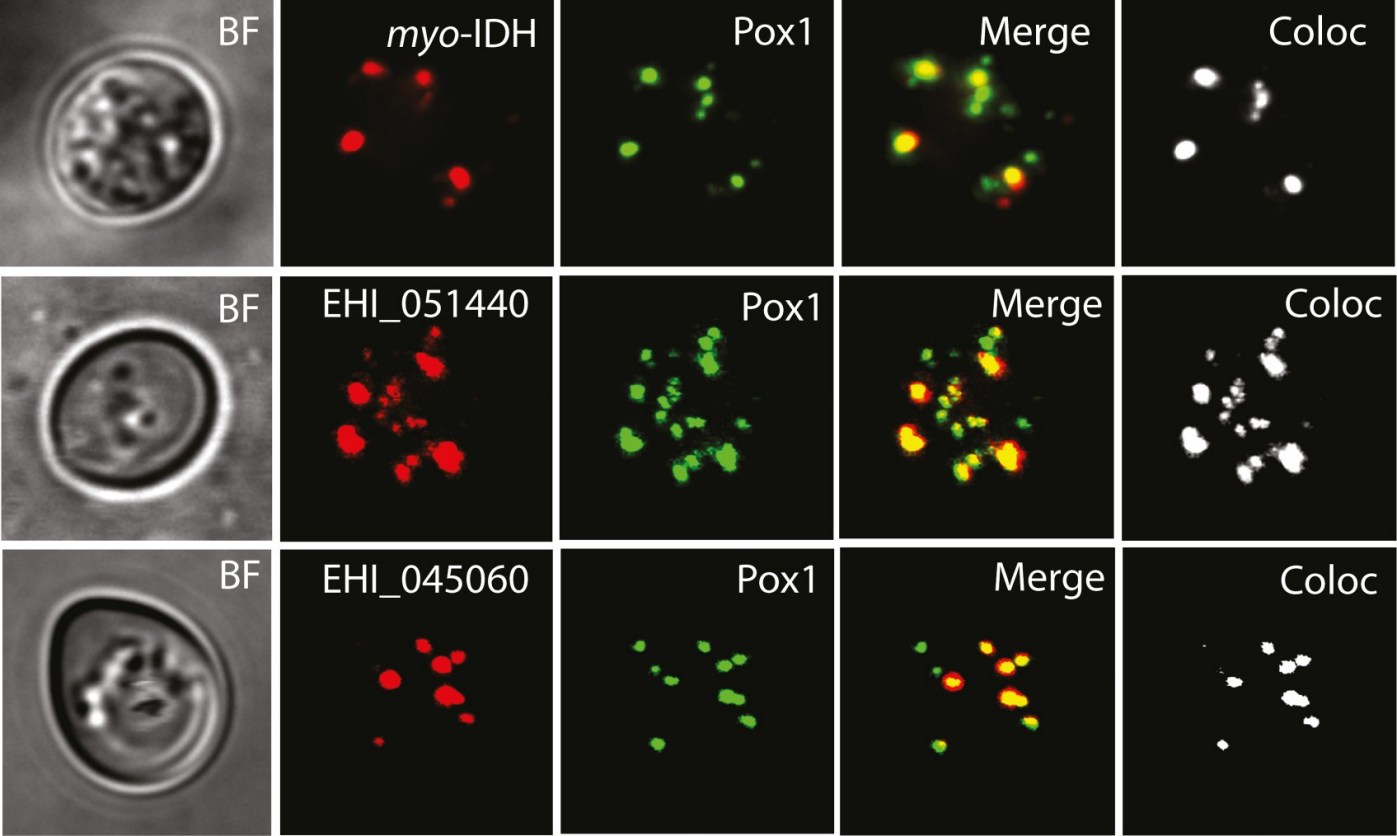
Localization of PTS1-containing candidate proteins in yeast peroxisomes. Fluorescence microscopy of mCherry-tagged *myo*-IDH and two hypothetical proteins (EHI_051440 and EHI_045060) (red) expressed in yeast. Pox1 fused with GFP was used as a peroxisomal marker (green). Signal colocalization analysis (white) was performed by ImarisColoc software.

**Fig 10:**
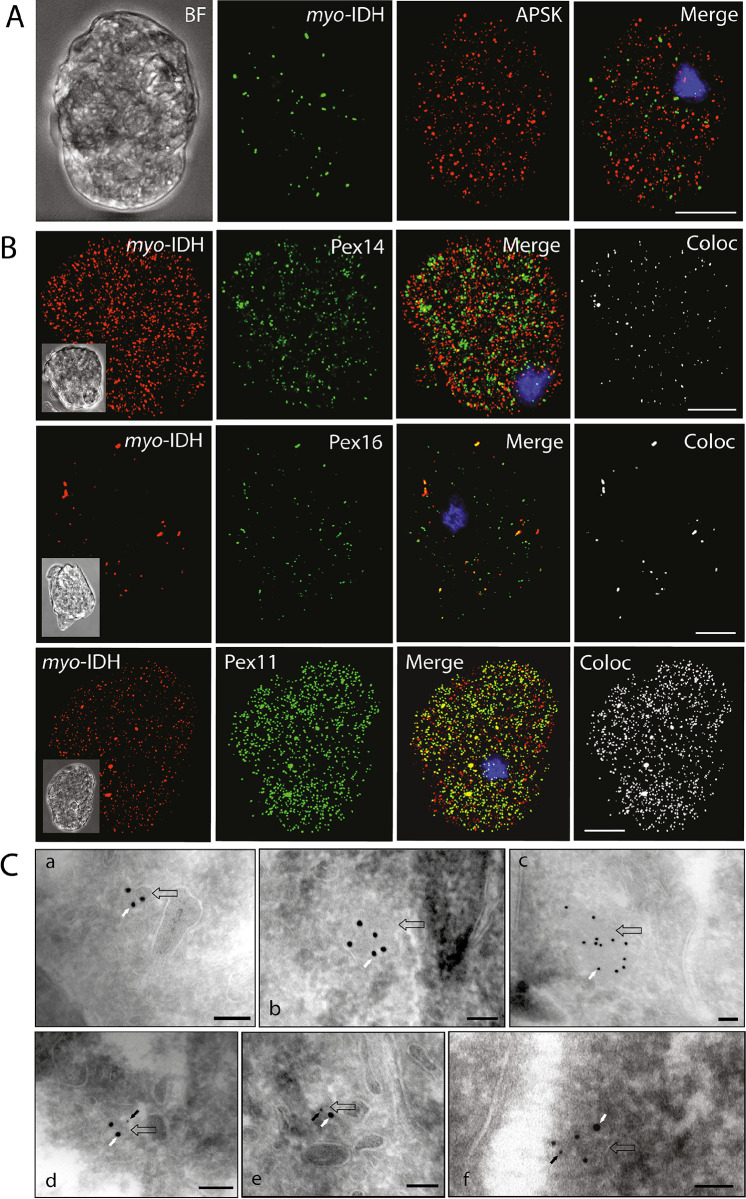
Localization of IDH in *E*. *histolytica*. A. Detection of *myo*-IDH (green)and APSK (mitosomal marker protein, red) in wild- type cells. Bar = 10 μm. B. Detection of native *myo*-IDH (red) in cells expressing C-terminally His-tagged PEXs (green). Signal colocalization analysis (white) was performed by ImarisColoc software. Bar = 10 μm. C. Immunoelectron microscopy. *Myo*-IDH was detected using a rabbit polyclonal anti-*myo*-IDH antibody and anti-rabbit IgG conjugated to 15 nm gold particles (white arrows, a-f), Pex16 was detected using a mouse monoclonal anti-poly-His antibody, and anti-mouse IgG conjugated to 5 nm gold nanoparticles (black arrows, d-f). Empty arrows indicate membranes. Bars 100 μm.

Predicted peroxisomal localization was evaluated for seventeen selected proteins (S7 Table). The proteins were expressed with an N-terminal mCherry tag in the yeast strain BY4742:POX1-EGFP expressing the integrated GFP-tagged peroxisomal marker protein acyl-CoA oxidase (Pox1). Three proteins, putative IDH (Fig 9), and hypothetical proteins EHI_051440 and EHI_045060 were observed in round vesicles in which they colocalized with Pox1, whereas other localizations were observed for the rest of the tested proteins (Fig 9 and S7 Table). The localization of IDH in *E*. *histolytica* was studied using a specific polyclonal antibody raised against the corresponding recombinant protein (S4 Fig). Confocal immunofluorescence microscopy revealed IDH-labeled punctual structures distinct from mitosomes visualized by an antibody against APSK (PCC r = -0.159)(Fig 10A). Furthermore, we investigated the colocalization of IDH in cells expressing His-tagged PEXs (Figs 10B and S2 and S5 Tables). IDH was detected in similar structures as above and partially colocalized with Pex14 (PCC *r* = 0.512, Fig 10B) and Pex16 (PCC *r* = 0.260). The strongest correlation (PCC *r* = 0.547) was observed between IDH and Pex11 (Fig 10B). The vesicular localization of IDH was confirmed by immunoelectron microscopy (Fig 10C). The double-labeling experiments revealed IDH signals with those of Pex16 within the vesicular structures(Fig 10C).

**Fig 11:**
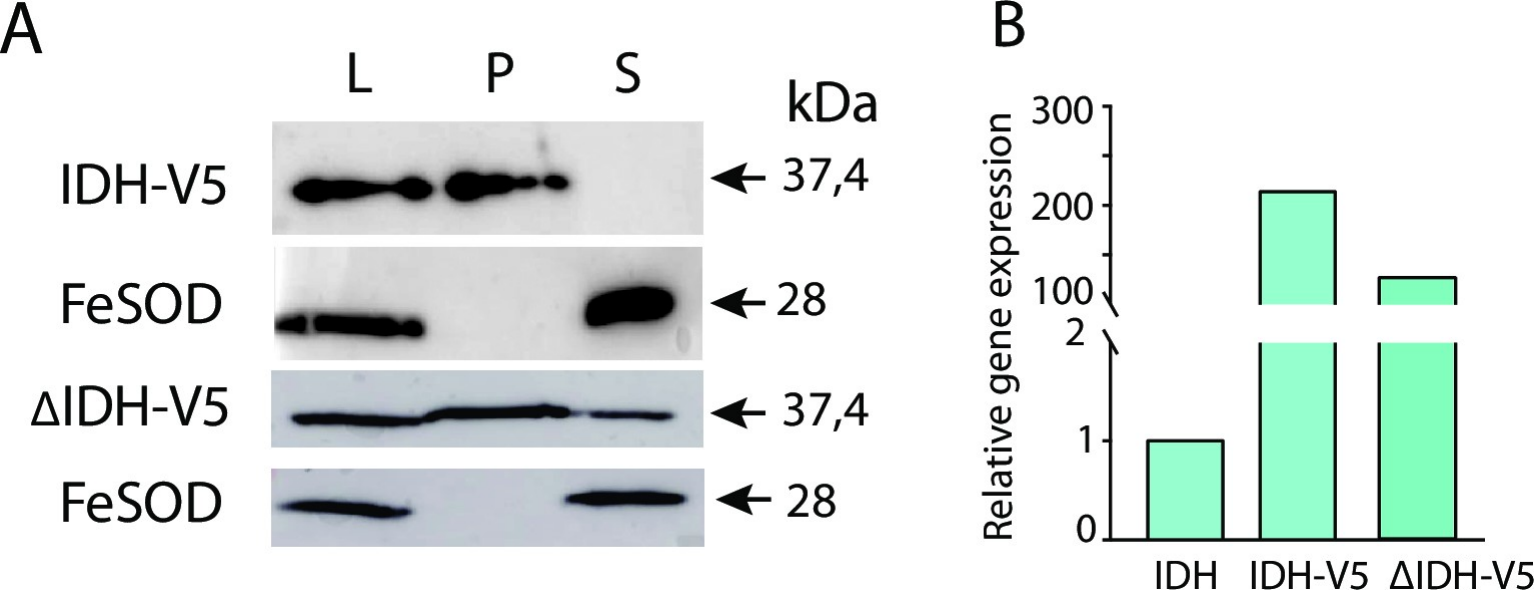
Effect of *myo*-IDH PTS1 tripeptide deletion. A. Detection of N-terminally V5-tagged full-length *myo*-IDH (IDH-V5) and its C-terminal truncated version (ΔIDH-V5) in the cell lysate (L), organelle (P) and cytosolic (S) fractions using western blot analysis. FeSOD was used as the cytosolic marker. B. Relative expression of IDH-V5 and ΔIDH-V5 using RT-qPCR. The level of native IDH was determined in wild-type cells and used for standardization.

Finally, we expressed IDH in *E*. *histolytica* with a V5-tag at the N-terminus. Expression of the gene was confirmed by RT-qPCR and by immunoblotting analysis of cell fractions (Fig 11). IDH was present exclusively in the sedimentable organellar fraction, while the cytosolic marker Fe-SOD was present in the soluble fraction. Next, we prepared a truncated version of the protein lacking C-terminal PTS1 tripeptide. The truncated gene was transcribed at a comparable level as the complete V5-tagged gene (Fig 11B). The deletion of PTS1 tripeptide resulted in dual localization of IDH in the cytosol and the organellar fraction (Fig 11A).

Collectively, the identification of IDH in the proteome of the putative peroxisome-enriched fraction, prediction of the PTS1 signal at the C-terminus with high statistical support, and localization studies in yeast and *E*. *histolytica* indicate that IDH is targeted to the matrix of *E*. *histolytica* organelle that contains PEXs, hereafter peroxisomes.

### Peroxisomal IDH preferentially utilizes *myo*-inositol as a substrate

Identification of putative IDH in peroxisomes prompted us to investigate the substrate specificity of this enzyme. An interproscan search revealed the presence of the N-terminal NAD-binding Rossmann fold domain of the glucose–fructose oxidoreductase (GFO)_IDH_MocA protein family (PFAM: PF01408). These enzymes can utilize a broad spectrum of substrates, such as *my*o-inositol, *scyllo-*inositol, D-glucose, D-xylose, D-fructose, and 1,5-anhydro-D-fructose [43–47]. Protein sequence alignment of *E*. *histolytica* IDH to IDHs of other archamoebae and structurally characterized bacterial paralogs revealed the presence of conserved residues required for the binding of NAD^+^ (motifs I and II) and key residues of motifs III-VI that define the substrate-binding pocket (S3 Fig) [48]. However, diversity within motif IV did not allow reliable estimation of the enzyme substrate specificity (S3 Fig). Thus, recombinant *E*. *histolytica* IDH was expressed in *E*. *coli*, affinity purified (S4 Fig), and used to determine its kinetic parameters for various substrates (Table 1 and S5 Fig). The enzyme revealed dehydrogenase activity preferentially toward *myo*-inositol (hereafter *myo-*IDH), and this activity was dependent on NAD^+^, while negligible activity was detected with NADP^+^. The Michaelis constant K_m_ for *scyllo*-inositol was approximately 10-fold higher than that for *myo*-inositol and those for D-glucose and D-xylose two orders of magnitude higher. The molecular mass of recombinant *myo*-IDH under reducing conditions using SDS-PAGE was 37.4 kDa, which corresponded well with the theoretical weight of 36.4 kDa, including the 6x-His tag. Molecular mass of the native enzyme was initially determined by size-exclusion chromatography. The peak of enzymatic activity was recovered at the elution volume corresponding to 41 kDa suggesting a momomeric structure (S6 Fig). However, because molecular mass determined by this approach might be affected by the protein shape, we also used multiangle light scattering (MALS). This analysis revealed that the molecular mass of the native recombinant enzyme was 77.4 (±4) kDa, suggesting a homodimeric structure with a minor contribution from the trimer (118.1± 22 kDa) (S7 Fig).

**Table 1:** Kinetic parameters of *E*. *histolytica myo*-IDH.

Substrate	K_m_^Substrate^ (mean±SD) [mM]	V_max_ (mean±SD) [μmol.min^-1^.mg^-1^]
*myo*-inositol	0.044±0.004	5.451±0.456
NAD^+^(*myo*-inositol)*	0.038±0.004	1.002±0.017
keto-2-inositol	0.322±0.034	4.409±0.053
NADH	0.009±0.000	1.638±0.024
*scyllo*-inositol	0.229±0.020	6.204±0.477
D-glucose	8.576±1.234	4.782±0.174
D-xylose	13.223±1.900	4.582±0.095
NADP^+^(*myo*-inositol)*	1.201±0.532	0.826±0.113

*12.5 mM *myo*-inositol. Lineweaver-Burk plots are given in S5 Fig.

### Peroxisomal IDH formed a monophyletic clade in phylogenetic reconstruction

**Fig 12:**
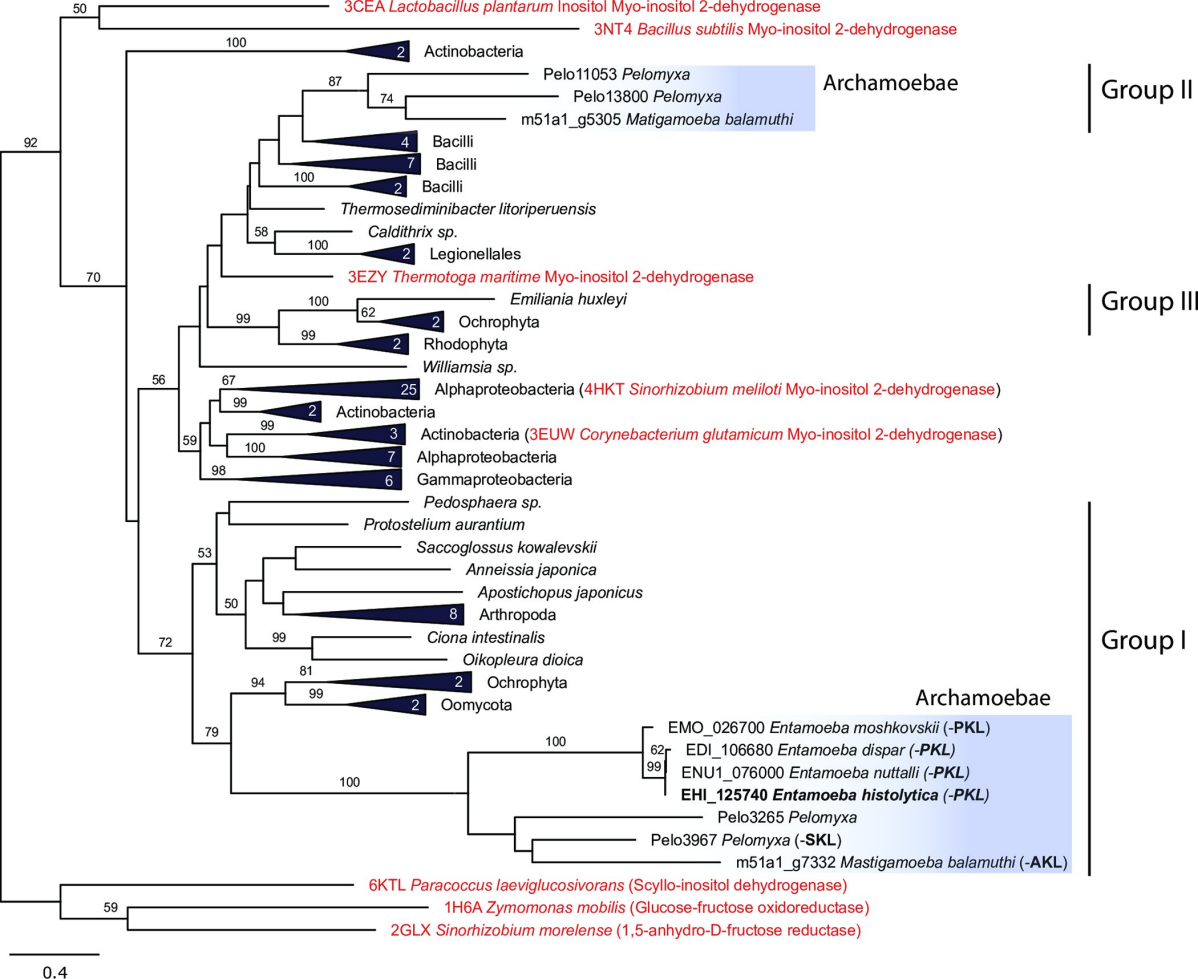
Phylogeny of *myo*-IDH. The maximum likelihood tree was inferred with IQ-TREE using 103 protein sequences and 289 positions. Bootstrap support is given at nodes. Sequences with known crystal structures are highlighted in red. Numbers in triangles indicate the number of included sequences. PTS1 triplet is given in brackets.

To investigate the evolution of *E*. *histolytica myo*-IDH, we performed phylogenetic analysis using eukaryotic (33) and prokaryotic (70) protein sequences, including those with known structures and functions (Figs 12 and S8). The eukaryotic sequences formed three distinct branches nested within bacterial orthologs. The IDH sequences identified in four *Entamoeba* species, *M*. *balamuthi* and *P*. *schiedti*, formed a monophyletic cluster of peroxisomal IDHs with high statistical support. All these sequences possessed PTS1 signals (-PKL, -SKL, -AKL), except for one of two *P*. *schiedti* IDH paralogs. The peroxisomal IDHs were placed within broader mostly eukaryotic branches (Group I). The closest orthologs appeared to be putative IDHs in ochrophytes and oomycotes, and we also identified IDH orthologs in the amoebozoan *Planoprotostelium fungivorum* and members of Hemochordata, Tunicata, Echinodermata, and Arthropoda. None of these orthologs possessed a PTS1 signal, although a rare PTS1 signal was noted for the IDH of *P*. *fungivorum*(-SKF). The cellular localization of most eukaryotic IDHs of Group I is likely cytosolic; however, IDHs of Stramenopiles that formed a sister group to peroxisomal IDHs were predicted to localize to mitochondria. In addition, IDHs of red algae with primary plastids and the haptophyte *Emiliania huxleyi* and two members of Ochrophyta with secondary plastids of red algal origin were predicted to target chloroplasts [49] (Group III) (S8 Table).

Collectively, the evolutionary history of eukaryotic IDHs appeared to be rather complex. The observed topology suggests at least three independent lateral gene transfers of IDHs from bacteria to eukaryotes as well as possible transfer between eukaryotes via plastids of the red lineage. Peroxisomal *myo*-IDHs seem to have evolved from a common ancestor of Archamoebae independent of cytosolic IDH homologs. In *E*. *invadens*, *myo*-IDH was most likely secondarily lost.

## Discussion

*E*. *histolytica* is believed to be devoid of peroxisomes, like most anaerobic protists [26,50]. In this work, we provided the first evidence that peroxisomes are present in this parasite, although *E*. *histolytica* peroxisomes seem to be remarkably reduced in comparison to their known peroxisome counterparts. *E*. *histolytica* contains only seven homologs of known PEXs (Pex1, Pex6, Pex5, Pex11, Pex14, Pex16, and Pex19) that may participate in organelle biogenesis. Targeting of matrix proteins to peroxisomes is reduced to the PTS1-dependent pathway mediated via the soluble Pex5 receptor, while the PTS2 receptor Pex7 is absent. Proteomic analyses of affinity-purified peroxisomes and *in silico* PTS1 predictions led to the identification of peroxisomal *myo*-IDH. Importantly, *E*. *histolytica myo*-IDH with PTS1 was shared with *E*. *dispar*, *E*. *nutalli* and *E*. *moshkovskii* and with evolutionarily related free-living *M*. *balamuthi* and *P*. *schiedti*, which all belong to the Archamoebae group of anaerobic protists, whereas *E*. *invadens* most likely lost peroxisomal *myo*-IDH together with peroxisomes.

The presence of peroxisomes in *E*. *histolytica* is supported by the localization of Pex5, Pex11, Pex14, Pex16, and Pex19 to numerous vesicles that are distinct from the structures of the ER, endosomal/lysosomal vesicles and mitosomes. Pex14 and Pex16 were detected in single membrane-bound vesicles of approximately 90–100 nm in diameter, which is within the range of anaerobic peroxisomes (80–440 nm) observed in *M*. *balamuthi* [27]. The matrix protein *myo*-IDH colocalized with Pox1 in yeast peroxisomes and within organelles labeled with Pex14, Pex16, and Pex11 in *E*. *histolytica*. Deletion of PTS1 tripeptide caused partial mistargeting of *myo*-IDH to the cytosol.

There are multiple metabolic interplays between mitochondria and peroxisomes, and in some cases, mitochondria may participate in peroxisome biogenesis [25]. However, the metabolism of *E*. *histolytica* mitochondria (mitosome) is reduced to a single pathway, and their protein import machinery contains only 2 out of 30 components known in model yeast mitochondria [9,51,52]. In this view, the observed reduction in peroxisomes is consistent with overall changes during the course of reductive evolution that formed *Entamoeba* species [5]. Most eukaryotes possess 13–17 PEXs, including humans and *M*. *balamuthi*, and four PEXs, Pex3, Pex10, Pex12, and Pex19, are considered peroxisomal markers absent only in organisms devoid of peroxisomes [53]. Three of these markers (Pex3, Pex10, and Pex12) were not identified in *E*. *histolytica* (Fig 1A). Pex3, together with Pex16 and Pex19, are involved in peroxisomal membrane protein (PMP) import. Pex3 facilitated the membrane docking of newly synthesized PMPs delivered via the shuttling receptor Pex19 directly from the cytosol (class I pathway for PMP import). Pex3 itself and other PMPs are recruited to the ER membrane by Pex16, which is cotranslationally inserted into the ER and subsequently delivered to peroxisomes through vesicular transport (class II pathway) [25,38]. We speculate that Pex16 might be sufficient for PMP import without a Pex3 contribution in *E*. *histolytica*. In support of this possibility, the formation of membrane structures with PMPs was observed in cells in which *pex3* was deleted [54], and the budding of pre-peroxisomal vesicles was dependent on Pex19, a homolog of which we found in *Entamoeba* [55]. Moreover, the presence of Pex16 and Pex19 without Pex3 was noticed in *Tetrahymena thermophila* [50]. Nevertheless, we cannot rule out the possibility that *E*. *histolytica* possesses a highly divergent Pex3 that was not recognized by current bioinformatic tools. Indeed, the highly divergent trypanosomal Pex3 remained elusive for a long time, and it has been identified only recently [56,57]. However, the absence of the N-terminal domain of *E*. *histolytica* Pex19, which is essential for its interaction with Pex3 [30,31] is more consistent with the lack of Pex3 in this organism. Interestingly, Pex19 is the only peroxin, homolog of which we identified in *E*. *invadens* that likely lack peroxisomes. Thus, Pex19 may have a more general function in protein sorting, which is unrelated to peroxisomes. Pex10 and Pex12 (RING complex) belong to the Zn-RING finger E3 ubiquitin-protein ligase family that, together with the E2 conjugating protein Pex4, participate in Pex5 recycling.Pex5 with peroxisomal cargo associates with membrane-docking components to form a transient pore with Pex14. Upon the release of the cargo, Pex5 is dislocated from the membrane in an ATP-dependent manner by Pex1 and Pex6 (dislocase complex) [58], which we both identified in *E*. *histolytica*. Recognition of Pex5 by the dislocase complex is dependent on Pex5 ubiquitination via thiol ester bond at the conserved cysteine at the N-terminus by the RING complex [59]. The absence of Pex10, Pex12, and Pex4 in *E*. *histolytica* suggests that Pex5 might be recycled without a ubiquitination step. In support of this view, *E*. *histolytica* Pex5 lacks an N-terminal cysteine residue. Moreover, in *Pichia pastoris*, PTS receptors and Pex14 have been shown to form the minimal translocation machinery that can facilitate peroxisomal import independent of the RING complex [60]. Alternatively, other E3 and E2 family proteins may compensate for the absence of the RING complex and Pex4. *E*. *histolytica* possesses genes for at least six E3 ligases, of which the E3 ligase EHI_030770 was found in the proteome of the peroxisome-enriched fraction, although, unlike Pex10 and Pex12, EHI_030770 does not possess any predicted transmembrane domain (S7 Table). There are also two E2 ubiquitin conjugating (Ubc) proteins (EHI_083560, EHI_048700) in the proteome, of which EHI_083560 (named EhUbc5) was partially characterized and crystalized, however, its target proteins are not known [61]. In metazoans and fungi, Pex4 is absent and its function is compensated by Ubc-conjugating proteins [62,63]. However, replacement of Pex10 and Pex12 has not been observed thus far. In contrast, the absence of the PTS2 receptor Pex7 observed in *E*. *histolytica* is not unprecedented, and when Pex7 is present, PTS2 targets peroxisomes much less frequently than PTS1s [64]. For example, Pex7 was found in related *M*. *balamuthi* in which PTS2 was predicted in 15% of putative peroxisomal proteins [27]. The absence of Pex7 was noticed in nematodes, several arthropods, the rhodophyte *Galdieria sulpharia*, and the stramenopile *Thalassiosira pseudonana* [50,65].

Interestingly, *E*. *histolytica* Pex11 displayed dual localization in peroxisomes and mitosomes. Previously, mitochondrial localization of Pex11 was observed only in cells such as yeast and human fibroblasts that lack peroxisomes upon deletion of Pex3 and Pex19 [66,67]. It seems that Pex11 has some affinity to mitochondria, possibly via mitochondrial proteins, with which Pex11 interacts. Pex11 is involved in elongation and proliferation of peroxisomes, which share with mitochondria dynamin-related proteins (DRPs) for the peroxisomal division; Pex11 can interact with mitochondria via the ERMES complex [66] and the translocase of the outer mitochondrial membrane (TOM) complex receptor Tom22 [68]. Neither ERMES nor Tom22 are present in mitosomes of *E*. *histolytica* [69,70]; however, *E*. *histolytica* possesses two DRPs that are involved in the fission of mitosomes and may potentially interact with Pex11 [71]. Interestingly, Pex11 was previously identified in the mitosomal proteome, supporting its dual localization [9]. However, these proteomic data need to be considered with caution, as in our experiments, peroxisomes and mitosomes comigrated using standard density gradient separation and it is likely that peroxisomes contaminated the mitosomal proteome. We cannot rule out the possibility that the partial mitosomal localization of Pex11 was a result of its mislocalization due to protein overexpression. However, overexpression of Pex11 in *E*. *histolytica* estimated by RT-qPCR was the lowest compared to four other recombinant PEXs for which no association with mitosomes was observed.

The matrix of *E*. *histolytica* peroxisomes contains *myo*-IDH, which catalyzes reversible NAD^+^-dependent conversion of *myo*-inositol to keto-2-inositol. *Myo*-inositol is synthesized from glucose-6-phosphate by the activity of inositol 3-phosphate synthase and inositol-3-phosphatase, which are both present in *E*. *histolytica*. *Myo*-inositol is utilized for the synthesis of phosphatidylinositol and a spectrum of phosphoinositide derivatives that are produced by a set of phosphatidylinositol kinases and phosphatases [72,73]. These compounds have multiple roles as components of membrane lipids, they are used in cell signaling pathways, energy homeostasis, and as cytoprotective solutes [74,75]. In mammals, insects, yeast and Chlorophyta, the catabolism of *myo*-inositol is initiated by oxygen-dependent *myo*-inositol oxygenase that converts *myo*-inositol to glucuronic acid [76]. However, none of these eukaryotic biosynthetic or catabolic pathways required the activity of *myo*-IDH. Bacteria such as *Klebsiella aerogenes* can grow on *myo*-inositol and other cyclitols as sole carbon sources. Here, *myo*-IDH catalyzes the first step of the catabolic pathway, leading to the production of acetyl-CoA [77]. However, our searches did not identify any enzyme downstream from *myo*-IDH in this pathway in *E*. *histolytica*. The distribution of *myo*-IDH in eukaryotes is limited to a few lineages of algae [78] and invertebrates. The function of *myo*-IDH remains enigmatic; however, algae contain two isoenzymes that were predicted to localize in the cytosol and mitochondria. It could be hypothesized that predicted dual localization may allow shuttling of a reducing power between mitochondria and the cytosol via myo-inositol/keto-2-inositol transport across the mitochondrial membrane [78]. Interestingly, dual localization of *myo*-IDH in peroxisomes and the cytosol was predicted in *M*. *balamuthi* and *P*. *schiedti*, suggesting the possibility of NAD-linked redox shuttling between the cytosol and peroxisomes to maintain intraperoxisomal redox as in the case of NAD^+^-linked malate dehydrogenase and glycerol-3-phosphate dehydrogenase-dependent shuttle systems that maintain the redox balance in yeast peroxisomes [79]. In Entamoeba species, we found only peroxisomal *myo*-IDH, which makes this explanation less plausible, although dual localization of proteins with peroxisomal targeting under specific physiological conditions has been observed [80]. Although the biochemical context of *E*. *histolytica myo*-IDH needs further investigation, the enzyme displayed two unusual features. First, *E*. *histolytica myo*-IDH has a notably higher affinity for *myo*-inositol characterized by a Km value that is three and four orders of magnitude lower (Km 0.044 mM) than that of the characterized orthologs in *Bacillus subtilis* (Km 18 mM) and the red alga *Galdieria sulphuraria* (Km 430 mM). Similar to the bacterial enzyme, D-glucose and D-xylose can serve as substrates for *E*. *histolytica myo*-IDH with Km values increased to 8.5 mM and 13.2 mM, respectively. These values are still two orders of magnitude lower than those in the bacterial enzyme (167 mM and 190 mM) [43,81]. Second, *E*. *histolytica myo*-IDH appeared to be highly active as a homodimer. In *B*. *subtilis* and *G*. *sulphuraria*, the molecular weight of native *myo*-IDH was approximately 160 kDa, which indicated a homotetrameric structure, which has been confirmed by structural studies in *B*. *subtilis* [48] and *Lactobacillus casei* [82].

In conclusion, findings of peroxisomes in *E*. *histolytica* and previously in *M*. *balamuthi* erode the paradigm of peroxisome absence in anaerobes. It also suggested that a minimal set of only seven peroxins might be sufficient to build these organelles. Moreover, the specific parasite peroxisomes with a functional *myo*-IDH that is absent in host cells might be an interesting target for the development of antiparasitic drugs. However, this report represents only a starting point for further functional investigations of anaerobic peroxisomes in this important human parasite.

## Materials and methods

### Searches for peroxisomal proteins

Profile hidden Markov models (HMMs) of PEXs (Pex1, 2, 3, 4, 5, 6, 7, 10, 11, 12, 13, 14, 16, 19, 26) were collected from the EggNOG database of orthologous groups (version 5.0) [83]. Predicted proteins of *E*. *histolytica* were searched for homologs of PEXs using HMMER (version 3.3) [84]. Putative PEXs were analyzed by searching against the NCBI nr protein database using BLAST [85], EggNOG database using HMMER, and the Pfam and COG databases using HHpred [86]. PTS1 was predicted in the protein sequences of *E*. *histolytica* (https://amoebadb.org/amoeba/app) using a support vector machine with support vector classification (SVC)(scikit-learn.org). The model was trained using a dataset of 217 manually curated peroxisomal candidates selected from 11 amoebozoan proteomes, including experimentally verified peroxisomal proteins of *M*. *balamuthi* [27] with the omission of *Entamoeba* species (S1 Dataset). The predicted proteome of *Entamoeba invadens*, which lacks PTS1 receptors, served as the PTS1-negative set. The script and training data are available at https://github.com/vojtech-zarsky/PredictPTS1_ML. The SVC-based machine learning score (MLS) was calculated using ten C-terminal amino acid residues, and queries with a calculated MLS≥0.1 were predicted as PTS1. Three additional PTS1 prediction methods were used, including the PTS1 predictor at PeroxisomeDB (http://www.peroxisomedb.org/), PTS1 Predictor (https://mendel.imp.ac.at/pts1/) [42] and local searches for a strict consensus triplet motif (S/A/C)(K/R/H)L [87] and a relaxed motif (S/A/C/H/K/N/P/T)(K/R/H/N/Q/S)(L/I/M/F/A/V/Y) [88]. In a query sequence, the presence of amino acids of the strict motif was scored as 1, and any other amino acid residues in the relax motif were scored as 0.5. A sum of the triplet score TS ≥2.5 predicted PTS1.

### Phylogenetic analysis

Representatives of the ATPases associated with various cellular activities (AAA) and protein families with various functions were selected from the Swiss-Prot protein database (https://www.uniprot.org/) and aligned with predicted Pex1 and Pex6 amino acid sequences using MAFFT (version 7) [89]. Representatives of GFO_IDH_MocA protein family were selected from the Swiss-Prot protein database and known crystal structures [47,48]. The alignments were trimmed using Block Mapping and Gathering with Entropy (BMGE) [90], and the phylogenetic tree was estimated using IQ-TREE (version 1.5) [91] with the LG+I+G4 model. Bootstrap support values were calculated using 500 bootstrap replicates. The Bayesian inference of the phylogenetic tree was estimated using PhyloBayes with the CAT mixture model [92]. The alignments are available in Mendeley Data, doi: 10.17632/dsszxzcc84.1.

### Cultivation and transformation

*E*. *histolytica* cell line B2-5 derived from HM:IMSS was used for the study. The cell line was derived from Bernhard Nocht Institute of Tropical Medicine in Hamburg, Germany [93]. Cells were grown in TYI-S-33 medium [94] supplemented with 10% adult bovine serum (Merck/Sigma-Aldrich, St. Louis, MO, USA). *E*. *histolytica* transformations were performed using a lipofection protocol as described previously [95]. After transfection, *Entamoeba* cells were allowed to form a monolayer (approximately two days) before selection. Initially, 2 μg/ml G-418 or 1 μg/ml hygromycin was used, and after two days, the drug concentrations were increased to 20 μg/ml G-418 and 6 μg/ml hygromycin for at least one week of selection. The successful transformation was evaluated by real-time PCR (RT qPCR) and western blotting.

### RNA isolation and RT qPCR

Total RNA was isolated using TRI Reagent (Merck/Sigma-Aldrich, St. Louis, MO, USA) and quantified using a Nanodrop 2000B (Thermo Fisher Scientific, Waltham, MA, USA). DNAse I treatment was performed for total RNA (2 μg) according to the manufacturer’s instructions (Thermo Fisher Scientific, Waltham, MA, USA). cDNA amplification and RT-qPCR were performed in an RG-3000 cycler (Corbett Research/Qiagen, Hilden, Germany) using a one-step RT-qPCR kit (Merck/Sigma-Aldrich, St. Louis, MO, USA) as described [96]. Actin was used for normalization according to Meyer et al. [34]. The relative gene expression was measured using the 2^-ΔΔCq^ method, and the statistical analysis was performed with a two-tailed Student’s t test using GraphPad Prism Version 7.0 [97]. All the primers are listed in S9 Table. Each experiment was performed in biological triplicates with technical duplicates.

### Gene cloning and expression

Genes coding putative Pex5 (EHI_179030), Pex11 (EHI_103470), Pex14 (EHI_194840), Pex16 (EHI_024620), Pex19 (EHI_198710) and *myo*-IDH (EHI_125740) were amplified using specific primers (S9 Table) and cloned without stop codons into the pNeoCass vector [34] via *Kpn*I and *BamH*I. A 6xHis tag or *E*. *histolytica* codon-optimized V5-tag sequence flanked by *Bgl*II and *BamH*I was added at the C-termini of PEXs. For *myo*-IDH, an *E*. *histolytica* codon-optimized V5-tag (S9 Table) was added at the N-terminus of the gene flanked by *Kpn*I and *Bgl*II and cloned into pHygCass, a derivative of pNeoCass bearing hygromycin resistance cassette. A truncated version of the protein without three C-terminal amino acid residues (AAs) was prepared using PCR with the 3’ primer annealing to the920-939 bp position (S9 Table).

### Expression and localization of proteins in *S*. *cerevisiae*

Peroxisomal candidates were amplified and subcloned into a modified pTVU100 vector [98] that allows protein expression in yeast with an N-terminal mCherry tag (pTVU100-N-mCherry). *S*. *cerevisiae* BY4742:POX1-EGFP (kindly provided by Zdena Palková, Charles University, Czech Republic) was transformed with the pTVU constructs and selected as described [27]. Transformed strains were incubated for 15–20 h in oleate medium prior to microscopy to stimulate peroxisome formation.

### Cell fractionation

Cells (250 ml) were harvested [34], washed, and resuspended in MES isolation buffer (1 M sorbitol, 5 mM methanesulfonic acid, 1 mM KCl, 5 mM EDTA; pH 5.5). The pellet was resuspended in MES buffer containing inhibitors of proteases (0.5 mg/ml E-64, 10 μg/ml leupeptin, 50 μg/ml tosyl-L-lysyl-chloromethane hydrochloride [TLCK] and 1 tablet/50 ml Roche Complete Inhibitor Cocktail [Roche]) with or without 0.05% Tween-20. The mixture was homogenized using a Dounce homogenizer. The integrity of the cells was evaluated under microscopy, and homogenization proceeded until more than 95% of the cells were broken. Then, the homogenate was spun for 4 minutes at 380 x g to remove unbroken cells, and the supernatant was subjected either to organelle pull-down or differential centrifugation at 3,000, 9,000, 15,000, 50,000, and 150,000 x g steps (15 min per step). Alternatively, the supernatant was loaded on a 2 M sucrose-containing MES cushion and centrifuged at 50,000 x g for 20 min to separate soluble and sedimentable fractions.

### Affinity enrichment of peroxisomes

Peroxisomes were pulled down via C-terminally tagged Pex14 using a recombinant *E*. *histolytica* B2-5 strain expressing His-tagged or V5-tagged Pex14 and the untagged control. Cells were harvested and washed with NaPBS, and 10 mg of protein was used for pull-down experiments. The cells were resuspended in MES buffer supplemented with protease inhibitors with or without 0.05% Tween-20. The cells were homogenized on ice using a Dounce homogenizer, and the unbroken cells were removed. The cell lysate or the crude peroxisomal fraction (obtained from the homogenate ultracentrifugation at 150,000 x g for 15 min) was preincubated with magnetic beads containing non-specific mouse antibody (i. e. anti-His antibody for V5-tagged or anti-V5 antibody for His-tagged cells) to deplete the lysate of proteins binding nonspecifically; then, the precleared lysate was incubated with 50 μl of magnetic beads coupled with either anti-His or anti-V5 specific mouse antibody (MBL Life Science, Japan) for 90 min at 4°C on an overhead rotator. The beads were washed three times in 20 volumes of MES buffer. The proteins were identified by label-free quantitative-mass spectrometry.

### Mass spectrometry and data processing

Label-free quantitative mass spectrometry (LFQ-MS) was performed according to standard procedures as described previously [70]. Briefly, the samples were digested with trypsin, and the peptides obtained were subjected to nanoliquid chromatography-MS. The MS/MS spectra were acquired using Thermo Orbitrap Fusion (Q-OT- qIT, Thermo Fisher Scientific), processed in Proteome Discoverer 2.1. (Thermo Fisher Scientific) and searched against the *E*. *histolytica* database (downloaded from AmoebaDB.org, containing 8,306 entries). The quantifications were performed with label-free algorithms using MaxQuant software (version 1.6.2.1) [99], and the data were analyzed using Perseus 1.6.1.3 software [100].

### Dataset processing

Altogether 13 independent experiments were performed. The datasets were filtered using the following criteria: (i) the ratio of LFQ values for a given protein between cells expressing tagged Pex14 and wild-type cells was greater than 1, (ii) the protein was identified with at least 2 peptides, and (iii) the protein was identified in at least two independent experiments. The dataset from each experiment was sorted according to relative LFQ values, and each protein was scored: the protein with the highest relative LFQ was scored 100, and scores for other proteins were calculated successively [$a_{n}=100-(n-1)\times \frac{100}{x}$; x is the total number of proteins in a given dataset]. Annotations of the proteins were based on Release 51 (https://amoebadb.org/amoeba/app). The mass spectrometry proteomics data have been deposited to the ProteomeXchange consortium via the PRIDE partner repository [90] with identifier PXD026653 (http://www.ebi.ac.uk/pride/archive/projects/PXD026653).

### Production of recombinant *myo*-IDH and polyclonal antibodies

Recombinant 6xHis tagged *myo*-IDH was produced in *Escherichia coli* BL21 Rosetta cells (Novagen, Merck/Sigma-Aldrich, St. Louis, MO, USA) using the pET42b expression vector (Novagen, Merck/Sigma-Aldrich, St. Louis, MO, USA). The protein was purified by affinity chromatography using Ni-nitrilotriacetic acid agarose under denaturing conditions according to the manufacturer’s protocol (Merck/Qiagen, Hilden, Germany) and used to raise polyclonal antibodies in rats and rabbits (Davids Biotechnologie GmbH, Regensburg, Germany). Enzymatically active *myo*-IDH was expressed as above and affinity purified under native conditions using imidazole according to the manufacturer’s protocol (Merck/Qiagen, Hilden, Germany).

### Western blotting

Protein samples were separated by sodium dodecyl sulfate–polyacrylamide gel electrophoresis (SDS-PAGE), transferred to nitrocellulose, and probed using antibodies as indicated. Primary antibodies included mouse monoclonal anti-His [Merck/Qiagen, Hilden, Germany], mouse monoclonal anti-V5, 1:500 [Thermo Fisher Scientific, Waltham, MA, USA]; rabbit polyclonal anti-Cpn60, 1:1000 (a kind gift from T. Nozaki, Japan) antibodies; and rabbit polyclonal anti-*myo-*IDH, 1:500; and rabbit polyclonal anti-FeSOD antibodies, 1:1000 [101]. Anti-mouse or anti-rabbit horseradish peroxidase (HRP) conjugated secondary antibodies (Merck/Sigma-Aldrich, St. Louis, MO, USA) were used for detections. Visualization was performed with chemiluminescence (Immobilon Forte, Merck/Sigma-Aldrich, St. Louis, MO, USA) and images were obtained using ChemiDoc (BioRad, Hercules, USA).

### Immunofluorescence microscopy

Slides for confocal microscopy were prepared as described [51]. Briefly, cells were fixed using 3% formaldehyde in PBS for 30 minutes and then detached by gentle sonication in a water bath. Cells were pelleted, washed and permeabilized with 0.05% saponin followed by treatment with 50 mM NH_4_Cl. Upon washing, the cells were blocked using 2% inactivated fetal bovine serum in PBS with 0.05% saponin (FNS) followed by overnight incubation with primary antibody in a refrigerator (mouse monoclonal anti-His [Merck/Qiagen, Hilden, Germany], mouse monoclonal anti-V5, 1:500 [Thermo Fisher Scientific, Waltham, MA, USA]; rabbit polyclonal anti-adenosine phosphosulfate kinase [APSK], 1:1000; anti-BiP1, 1:500; and anti-Atg8, 1:1000 [a kind gift from T. Nozaki, Japan]; and rabbit and rat polyclonal anti-*myo-*IDH, 1:500). Alexa Fluor-488 (1:1000) or Alexa Fluor-594 (1:1000) secondary antibodies were used for visualization (Thermo Fisher Scientific, Waltham, MA, USA). Slides were observed using a Leica SP8 FLIM confocal microscope (50 optical sections of 150 nm each). Images were deconvolved using Huygens 19.04 software (SVI) and further processed with Fiji software [102] and the Imaris 9.7.2 Package for Cell Biologists (Bitplane AG, Zurich, Switzerland).Voxel-based colocalization was performed using ImarisColoc. Costes’s automatic thresholding was applied to the images in the Z stack [103], and PCC in colocalized volume was calculated. The number of labeled organelles observed by confocal microscopy (20 optical slices of 1 μm each) in *E*. *histolytica* using His-tagged Pex14 as a marker was determined per 25 cells using Icy software [104]. The number of peroxisomes was expressed per 100 μm^2^.

Structured illumination microscopy (SIM) was performed on a 3D N-SIM microscope (Nikon Eclipse Ti-E, Nikon, Japan) equipped with a Nikon CFI SR Apo TIRF objective (100x oil, NA 1.49) as described [105].

### Immunoelectron microscopy

Transmission electron microscopy was performed using *E*. *histolytica* cells overexpressing polyHis- or V5-tagged Pex14 and Pex16, and samples were processed as described previously [27]. The sections with V5-tagged Pex14 were blocked with 1% fish skin gelatin (FSG) and immunodecorated using mouse anti-V5 antibody (1:20, Invitrogen/Thermo Fisher Scientific, Waltham, MA, USA) and goat anti-mouse antibody conjugated with 5 nm gold nanoparticles (BBI Solutions) for 1 h in 1% fish skin gelatin. Samples with polyHis-tagged Pex14 were blocked in buffer containing 1% bovine serum albumin/0.05% Tween 20 in 0.1 M HEPES for 1 h at room temperature. Labeling was performed using Ni-nitrilotriacetic acid (Ni-NTA) conjugated with 5 nm gold nanoparticles (Nanoprobes, Yaphank, USA). Sections with polyHis-tagged Pex16 were treated as described above or with mouse monoclonal anti-polyHis antibody (IgG2A, Merck/Sigma-Aldrich, St. Louis, MO, USA), and protein A conjugated with 5 nm gold nanoparticles (1:50)(CMC Utrecht) in 1% fish skin gelatin. *Myo*-IDH was detected using rabbit polyclonal anti-*myo*-IDH antibody and anti-rabbit IgG conjugated to 15 nm gold particles (Nanoprobes, Yaphank, USA).

### Enzymatic assay

The activity of *myo*-IDH (EC 1.1.1.18) was measured spectrophotometrically at 340 nm and room temperature, as described [106]. The reaction mixture contained 1 mM NAD^+^ in 10 mM sodium pyrophosphate buffer, pH 9, 30 μg of the purified enzyme, and a substrate. The Km value was determined by measurement of the reaction velocity using *myo*-inositol (50 μM-25 mM), *scyllo*-inositol (370 μM-15 mM), D-glucose (5 mM-400 mM), D-xylose (5 mM-400 mM), and keto-2-inositol (12.5 μM—250 μM). For the Km of NAD^+^ and NADP^+^, 12.5 mM *myo*-inositol was used for both NAD^+^(50 μM—1 mM)and NADP^+^(500 μM—1.5 mM). Each measurement was performed in triplicates. The protein concentration was measured using the Lowry method. All chemicals were purchased from Sigma-Aldrich (Merck/Sigma-Aldrich, St. Louis, MO, USA).

### Size exclusion chromatography and multiangle light scattering (MALS)

The native molecular mass of recombinant, 6xHis-tagged *myo*-IDH was determined using size-exclusion chromatography Superdex 200 Increase 10/300 GL column connected to BioLogic DuoFlow system (BioRad, Hercules, USA). The column was calibrated with BioRad gel filtration standard mixture (cat # 1511901), and the running buffer was 10 mM sodium pyrophosphate used for the enzymatic assay. Fractions of 0.5 ml were collected for monitoring the *myo*-IDH activity in the elution profile.

For MALS analysis, samples of enzymatically active *myo*-IDH (0,5–2 mg/ml) were injected onto an Agilent Biosec-3 column (4.6x300 mm) at a flow rate of 0.3 ml/min in 50 mM phosphate buffer (pH 7.0) and 300 mM NaCl at 15°C. The column was coupled with static light scattering (miniDAWN, Wyatt Technology), differential refractive index (Shodex RI-501) and Agilent 1260 Infinity II UV (Agilent Technologies) detectors. Data were analyzed using ASTRA software (Wyatt Technology).

## Supporting information

S1 Fig Full-length Western blot images for analysis of PEXs associated with *E*. *histolytica* cell fractions (A, Fig 4; B, Fig 8; and C, Fig 11). (PDF)

S2 Fig 2D histograms from colocalization analysis for Figs 5, 6 and 10 using ImarisColoc in Imaris package. The frequency plot is shown on a logarithmic scale (the high frequencies are shown in red-to-yellow, low frequencies are shown in blue-to-violet. The number indicates border bins. Pearson correlation coefficientsin volume are given in S5 Table.
(PDF)

S3 Fig Protein sequence alignment of *E*. *histolytica myo*-IDH to IDH orthologs of other Archamoebae species and selected bacterial sequences of known crystal structure. (DOCX)

S4 Fig Purification of recombinant His-tagged *myo*-IDH using Ni-NTA column under native condition and western blot analysis of *E*. *histolytica* cellular fractions using polyclonal antibodies raised against purified *myo*-IDH. (PDF)

S5 Fig Lineweaver–Burk plots constructed for determination of kinetic parameters of *E*. *histolytica myo*-IDH (Table 1). Each point was calculated from three measurements, error bars represent standard deviations.
(PDF)

S6 Fig Determination of the native molecular mass of recombinant 6xHis-tagged IDH using size exclusion chromatography. Superdex 200 Increase 10/300 GL column connected to BioLogic DuoFlow system (BioRad) was used for the analysis.
(PDF)

S7 Fig MALS analysis of *myo*-IDH. Elution profile at three different concentrations, 2.0 mg/ml (red), 1.0 mg/ml (magenta) and 0.5 mg/ml (green), with the indicated molar mass.
(PDF)

S8 Fig Phylogenetic tree of IDHs including their accession numbers. (PDF)

S1 Table Accession numbers of identified PEXs in *Entamoeba* species. (XLSX)

S2 Table Protein sequence identity of Entamoeba PEXs. (XLSX)

S3 Table Relative expression of PEXs and three housekeeping enzymes in *E*. *histolytica* determined by qRT-PCR. (DOCX)

S4 Table Relative expression of *PEX* genes in *E*. *histolytica* transformants overexpressing PEX. (DOCX)

S5 Table Pearson correlation coefficient (PCC) in colocalized volume calculated for images in Fig 5, Fig 6 and Fig 10. Voxel-based colocalization with automatic thresholding was performed using ImarisColoc. Corresponding 2Dhistograms are given in S1 Fig.
(DOCX)

S6 Table Proteins identified in peroxisomal fractions of *E*. *histolytica* by mass spectrometry. (XLSX)

S7 Table List of predicted peroxisomal proteins based on PTS1 predictions. (XLSX)

S8 Table Cellular localization predictions of putative inositol dehydrogenases. (XLSX)

S9 Table List of primers. (XLSX)

S1 Dataset The training set of amoebozoan peroxisomal proteins for PTS1 prediction using a support vector machine with support vector classification. (TXT)
