# Supplementary material for: Anaerobic peroxisomes in Entamoeba histolytica metabolize myo-inositol
Source: PLoS Pathog. 2021 Nov 15;17(11):e1010041. doi: 10.1371/journal.ppat.1010041 (PMC8629394; doi:10.1371/journal.ppat.1010041)

Fig S1. Full-length western blots

A. Expression of tagged PEXs in *E. histolytica*: full-length western blots presented in Fig 4A.

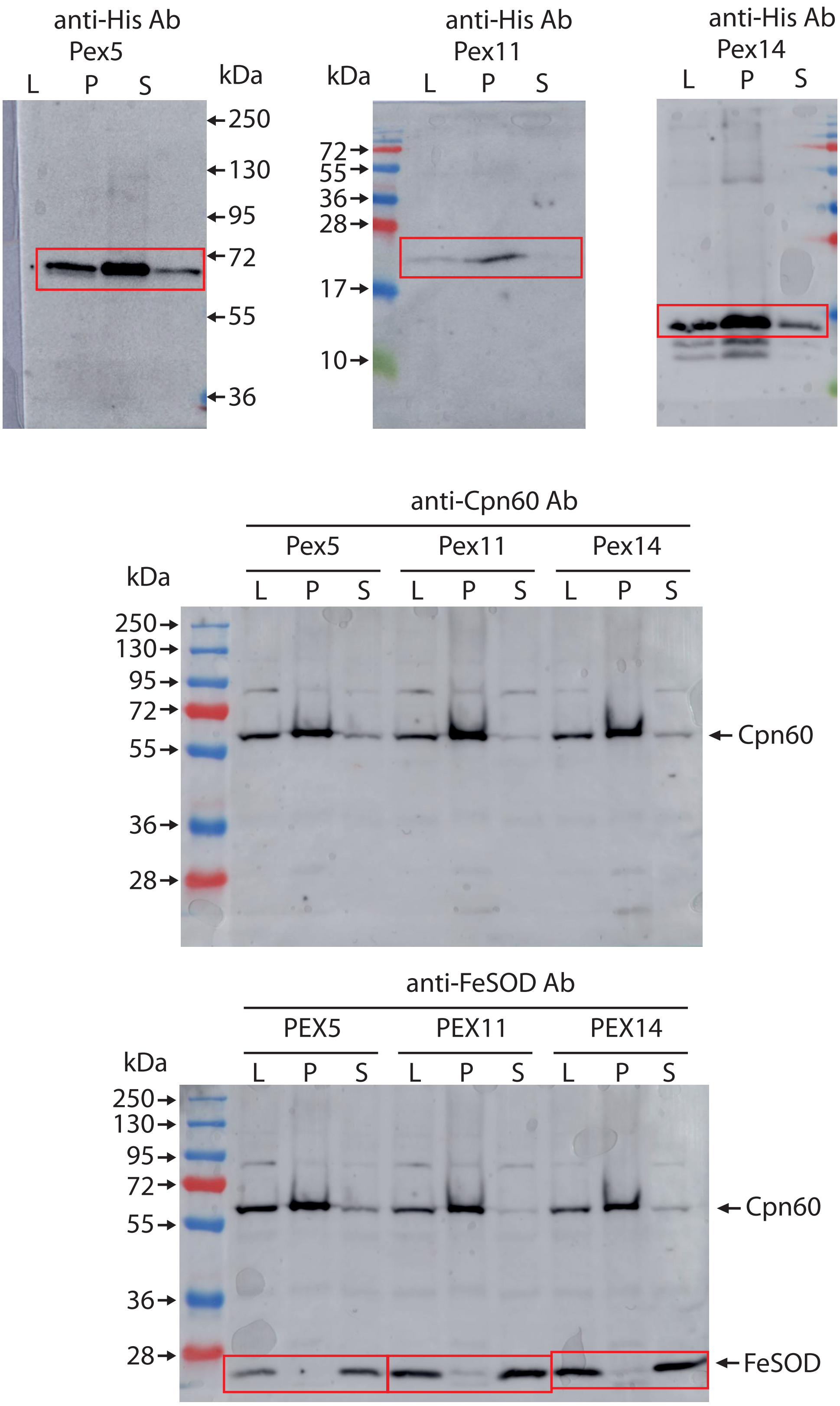

Samples of cellular fractions (L, lysate; P, pellet of organellar fraction; S, soluble fraction) were isolated from *E. histolytica* transfromants expressing N-terminally His-tagged Pex5, Pex11, and Pex14, separated by SDS PAGE, transferred to nitrocellulose and probed using mouse monoclonal anti-His primary antibody and anti-mouse HRP-conjugate secondary antibody (upper three blots). In parallel, the same samples were transblotted and probed with rabbit polyclonal anti-Cpn60 antibody (middle blot), and subsequently with rabbit polyclonal anti-FeSOD antibody (lower blot). The images were taken using ChemiDoc Imaging System, Bi-Rad. Red rectangles indicate cropped areas.

FigS1A. Expression of tagged PEXs in *E. histolytica*: full-length western blots presented in Fig 4A (continue)

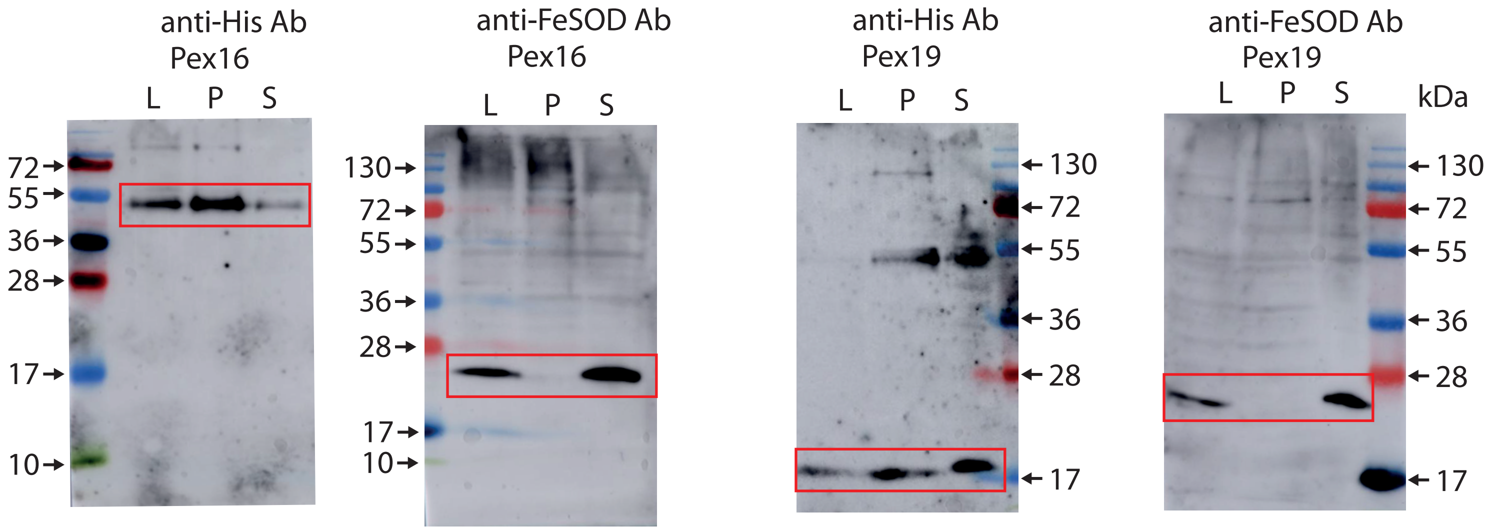

Samples of cellular fractions (L, lysate; P, pellet of organellar fraction; S, soluble fraction) were isolated from *E. histolytica* transfromants expressing N-terminally His-tagged Pex16, and Pex19, separated by SDS PAGE, transferred to nitrocellulose and probed using mouse monoclonal anti-His or rabbit polyclonal anti-FeSOD primary antibodies and anti-mouse/rabbit HRP-conjugate secondary antibodies.
The images were taken using ChemiDoc Imaging System, Bi-Rad. Red rectangles indicate cropped areas.

FigS1A. Expression of tagged PEXs in *E. histolytica*: full-length western blots presented in Fig 4B

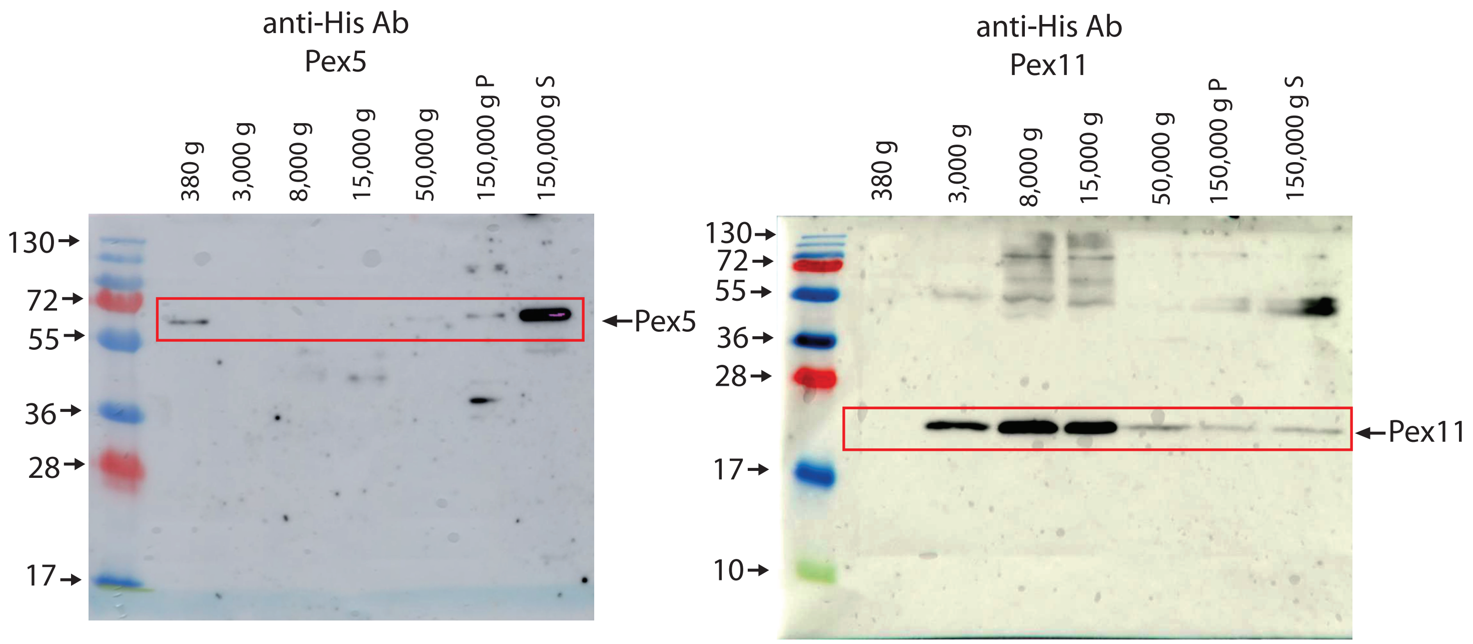

Western blot analysis of Pex5 and Pex11 in seven fractions isolated by differential centrifugation using 0.05% Tween-20 to limit protein aggregation. Red rectangles indicate cropped areas.

Fig S1B. Affinity purification of peroxisomes: blots are cropped in Fig 8.

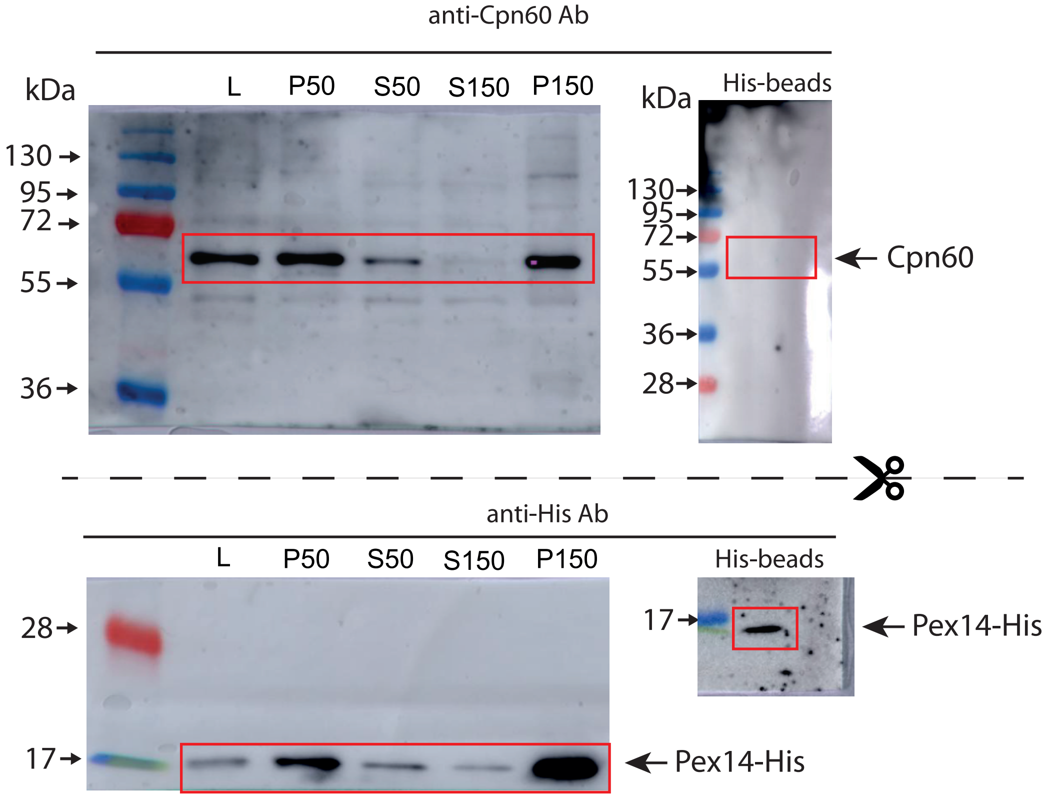

Lysate of cells (L) expressing His-tagged Pex14 was used for differential centrifugation at 50 000 and 150 000 x g, and pellet (P) and soluble (S) fractions were analyzed by western blotting. In the last step, peroxisomes were purified using anti-V5 antibody-conjugated beads (His-beads) that were used for mass spectrometry. Antibodies against Cpn60 were used as mitosomal markers. The samples were separated by SDS-PAGE, transferred to a nitrocellulose membrane, cut according to labeled molecular weight markers and probed with rabbit anti-Cpn60 and mouse anti-His primary antibodies (Ab), and anti-mouse/rabbit HRP-conjugate secondary antibodies. The images were obtained using ChemiDoc Imaging System, Bi-Rad. Red rectangles indicate cropped areas.

Fig S1C. Effect of *myo*-IDH PTS1 tripeptide deletion: Images of full-length blots which are cropped in Fig. 11.

Samples of lysate (L), pellet (P), and soluble (S) fractions isolated from *E. histolytica* expressing *myo*-IDH with N-terminal V5-tag and truncated C-terminal tripeptide.

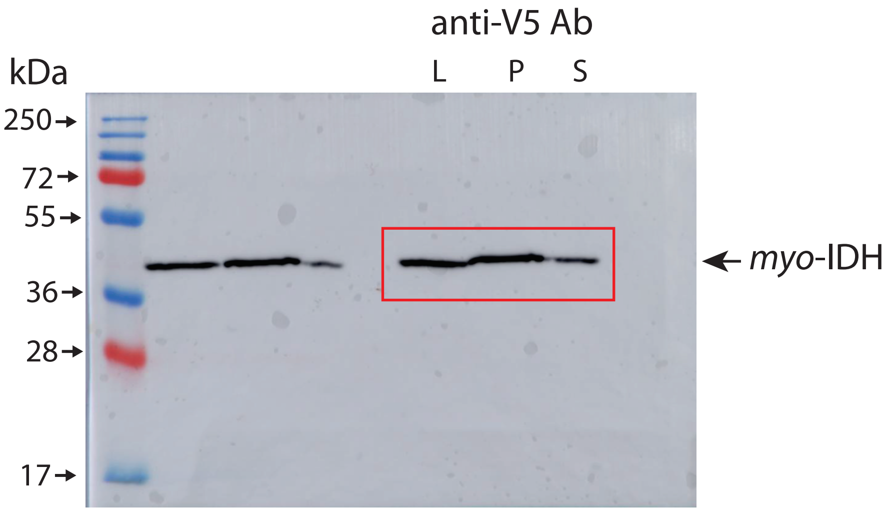

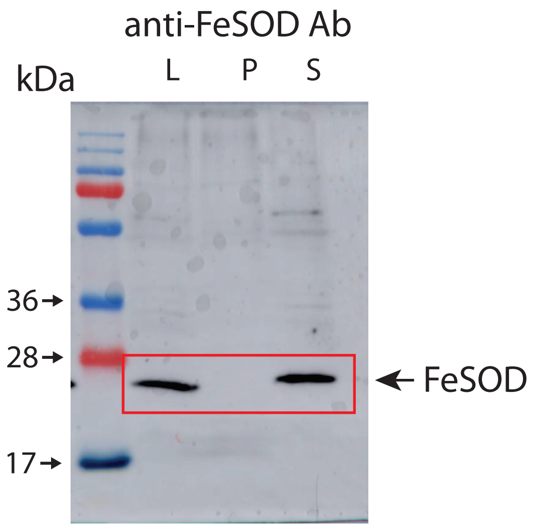

Samples of lysate (L), pellet (P), and soluble (S) fractions isolated from *E. histolytica* expressing *myo*-IDH with N-terminal V5-tag.

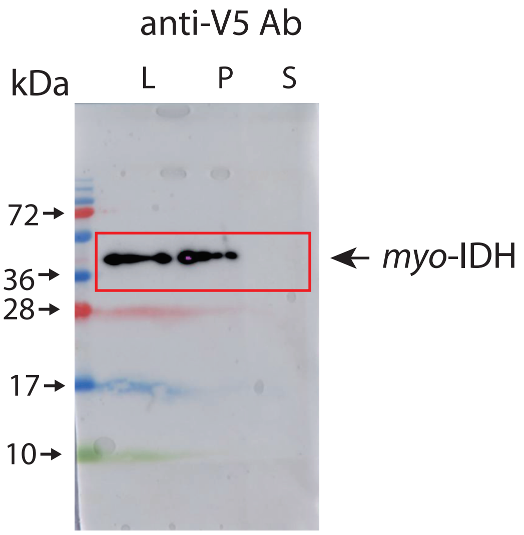

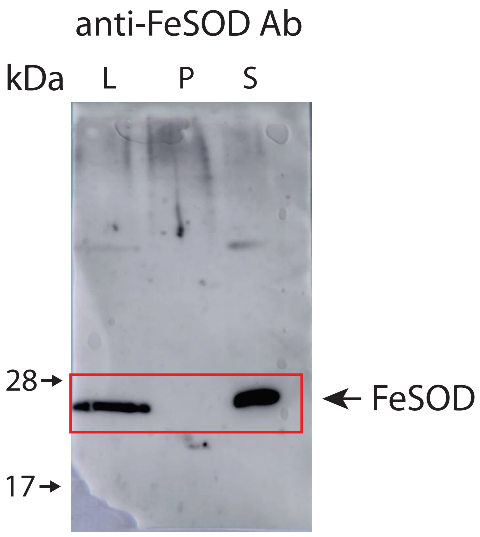

Supplement: S1 Fig — (PDF) [file ppat.1010041.s001.pdf]
