# Supplementary material for: Anaerobic peroxisomes in Entamoeba histolytica metabolize myo-inositol
Source: PLoS Pathog. 2021 Nov 15;17(11):e1010041. doi: 10.1371/journal.ppat.1010041 (PMC8629394; doi:10.1371/journal.ppat.1010041)
Supplement: S2 Fig — The frequency plot is shown on a logarithmic scale (the high frequencies are shown in red-to-yellow, low frequencies are shown in blue-to-violet. The number indicates border bins. Pearson correlation coefficientsin volume are given in S5 Table. (PDF) [file ppat.1010041.s002.pdf]

Fig S2. Histograms from ImarisColoc analysis corresponding to images in Fig 5, 6, and 10

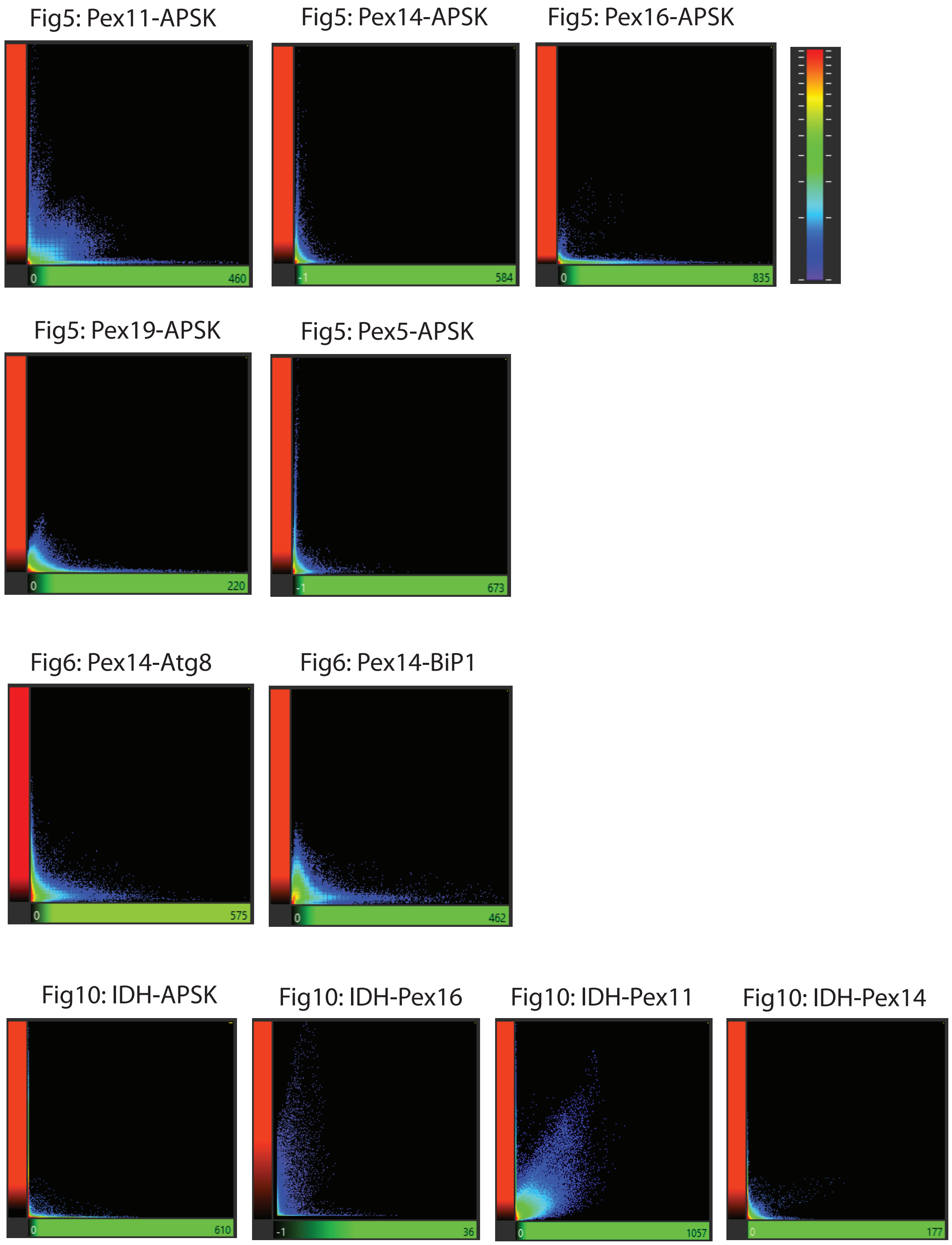

2D histograms from colocalization analysis for Fig 5, 6, and 10 using ImarisColoc in Imaris package. The frequency plot is shown on a logarithmic scale (the high frequencies are shown in red-to-yellow, low frequencies are shown in blue-to-violet. The number indicates border bins. Corresponding Pearson correlation coefficients are given in Table S5.
