# Supplementary material for: Anaerobic peroxisomes in Entamoeba histolytica metabolize myo-inositol
Source: PLoS Pathog. 2021 Nov 15;17(11):e1010041. doi: 10.1371/journal.ppat.1010041 (PMC8629394; doi:10.1371/journal.ppat.1010041)
Supplement: S3 Fig — (DOCX) [file ppat.1010041.s003.docx]

| *E. histolytica*  *MbIDH1*  *PsIDH1*  *PsIDH2*  *PsIDH3*  *PsIDH4*  MbIDH2  *B. subtilis (1)*  *S. typhimurium (1)*  *L.* casei *(2)*  *C.* tetani (*2)*  *R.* meliloti *(3)*  *T.* maritima (*3)*  *E.* coli *(4)*  *L. antri (4)*  *E. histolytica*  *MbIDH1*  *PsIDH1*  *PsIDH2*  *PsIDH3*  *PsIDH4*  MbIDH2  *B. subtilis (1)*  *S. typhimurium (1)*  *L.* casei *(2)*  *C.* tetani (*2)*  *R.* meliloti *(3)*  *T.* maritima (*3)*  *E.* coli *(4)*  *L. antri (4)*  *E. histolytica*  *MbIDH1*  *PsIDH1*  *PsIDH2*  *PsIDH3*  *PsIDH4*  MbIDH2  *B. subtilis (1)*  *S. typhimurium (1)*  *L.* casei *(2)*  *C.* tetani (*2)*  *R.* meliloti *(3)*  *T.* maritima (*3)*  *E.* coli *(4)*  *L. antri (4)*  *E. histolytica*  *MbIDH1*  *PsIDH1*  *PsIDH2*  *PsIDH3*  *PsIDH4*  MbIDH2  *B. subtilis (1)*  *S. typhimurium (1)*  *L.* casei *(2)*  *C.* tetani (*2)*  *R.* meliloti *(3)*  *T.* maritima (*3)*  *E.* coli *(4)*  *L. antri (4)*  *E. histolytica*  *MbIDH1*  *PsIDH1*  *PsIDH2*  *PsIDH3*  *PsIDH4*  MbIDH2  *B. subtilis (1)*  *S. typhimurium (1)*  *L.* casei *(2)*  *C.* tetani (*2)*  *R.* meliloti *(3)*  *T.* maritima (*3)*  *E.* coli *(4)*  *L. antri (4)*  β12  *E. histolytica*  *MbIDH1*  *PsIDH1*  *PsIDH2*  *PsIDH3*  *PsIDH4*  MbIDH2  *B. subtilis (1)*  *S. typhimurium (1)*  *L.* casei *(2)*  *C.* tetani (*2)*  *R.* meliloti *(3)*  *T.* maritima (*3)*  *E.* coli *(4)*  *L. antri (4)*  *E. histolytica*  *MbIDH1*  *PsIDH1*  *PsIDH2*  *PsIDH3*  *PsIDH4*  MbIDH2  *B. subtilis (1)*  *S. typhimurium (1)*  *L.* casei *(2)*  *C.* tetani (*2)*  *R.* meliloti *(3)*  *T.* maritima (*3)*  *E.* coli *(4)*  *L. antri (4)* | β1  α1  β2  α2  ---------- ---------- ------MQSN KVRICLCGTGRLGSFRASLF IE-SQDIELV GVVEP----- ---------F 39  ---------- ------MQSL ANDYPSAVAG TPRVVLFGFGRMGRFRFGLF DE-TGAVDCA AVVDPAMP-- ------GRSV 55  -----------------MASTTSSTTTTLT PVRLCQIGAGRIGTFRLGLF SR-SPHVRCV AVVESDR--- ------GNEV 53  MIDSELQSTC SNTGHHHHQI YGESPRDNDK LGRVCLVGAGRLGMNRLRLF SK--LGVPCT AVVEPNK--- ------SSPL 69  ---------- ---------- ---------- MINVAVIGGGRIGRLHLKNL SR-IPNVKVV GVAEIKPTED LYNLVAELAP 49  ---------- ---------- ------MQTT EVVLGVVGVGRIGRVHLENL LA-MHGVRVK TVCDWKILVD -----KEMME 48  ---------- ---------- ----MSTSSA PVVVGVIGVGRIGRMHIDNI LARLPAVRIA WVCDLLVDTD -----AEMRA 51  ---------- ---------- ---------M SLRIGVIGTGAIGKEHINRI TNKLSGAEIV AVTDVNQE-- ------AAQK 43  ---------- ---------- ---------M TLKAGIVGIGMIGSDHLRRL ANTVSGVEVV AVCDIVAG-- ------RAQA 43  ---------- ---------- ------MQTK TIKIGIVGLGRLGKIHATNI ATKIQHAKLQ AATSVVPA-- ------ELDW 46  ---------- ---------- ----MEENMN KIKVGIVGLGRLGRKHAENI ALRISNAELT AVCSVIKD-- ------EVDE 48  ---------- ---------- ---------M TVRFGLLGAGRIGKVHAKAV SG-NADARLV AVADAFPA-- ------AAEA 42  ---------- ---------- ---------- -MRIGVIGLGRIGTIHAENL KM-IDDAILY AISDVRED-- ------RLRE 40  ---------- ---------- --------MK KLRCGVIGLGRVGKMHVENM YL-LPQLDII CAADYFIE-- ------EMSD 43  ---------- ---------- -----MASKK RINAGIIGLGRAGQMHLKNL LT-IPEINIV QVSDIFVD-- ------KLAD 46  Motif I  α2  β3  RVC------- ---------- ---------- ---------- ---------- -------QSV L-DRPN---- ---------- 50  RAQ------- ---------- ---------- ---------- ---------- -------RRA V-DVPT---- ---------- 66  WAN------- ---------- ---------- ---------- ---------- -------SGK P-GHPT---- ---------- 64  WSE--PSIHK HPL------- ---GDRDAGV SEESAI---- ---------- ------GSSS A-SGDA---- ---------- 102  PLSRTPSPFR -PLSPTTSNG VCNGTPSTAT SLSSAVPICI ATTTPPLPSP SAPSPTPSVS V-TPPTPTTP HPTANGNGPV 127  WAV------- ---------- ---------- ---------- ---------- -------SRG F-TKFT---- ---------- 59  WLD------- ---------- ---------- ---------- ---------- -------ARA LGARRT---- ---------- 63  VVE------- ---------- ---------- ---------- ---------- -------QYQ LNATVY---- ---------- 55  ALD------- ---------- ---------- ---------- ---------- -------KYA IEAKDY---- ---------- 55  AKK------- ---------- ---------- ---------- ---------- -------ELG V-EEVF---- ---------- 57  AQK------- ---------- ---------- ---------- ---------- -------KLN V-KYGY---- ---------- 59  IAG------- ---------- ---------- ---------- ---------- -------AYG C-EVRT---- ---------- 53  MKE------- ---------- ---------- ---------- ---------- -------KLG V-EKAY---- ---------- 51  WLY------- ---------- ---------- ---------- ---------- -------SVN I-TSGY---- ---------- 54  QLN------- ---------- ---------- ---------- ---------- -------FMG I-TNQT---- ---------- 57  α5  β5  α3  α4  β4  ---------- ---------I KIFKTVEEI- -NIPIDGIWI STPTKFHLQT IKLASKK-VK HIYCEKPIAS TPEEVKEA-- 106  ---------- ---------- --FARLADV- PRASYDAVWV SCPTVHHPSA IRDALAAGVK MIYCEKMIAF DEEKVREC-- 121  ---------- ---------- -AFAALEDV- -NVQFDAVWI SCPTKYHPAM IEKAVNR-TR FVYCEKPIAY TAAQVKQC-- 118  ---------- ---------I HLYEDFSQV- PTSLYDGVWI ASSTASHPSC IGAASEK-TG NIFCEKPIAF DTSQVSSA-- 159  NGSRPTLSPT PSPPLAVLPF PIVEDYHTLL NDPTIEAVLV CTNTALHPQI CLDALKA-GK HVFCEKPVSY DLDVLRKL-- 204  ---------- ---------- ---TDYHEIT CDPEISAILI LSSTNSHVEI SIAAAEA-HK HIFCEKPVSS SLEEIETV-- 113  ---------- ---------- ---ADYREIL GDAAVEAVLV LCSTGAHLRI SLDALAA-RK HVFCEKPVSE SPAEIREV-- 117  ---------- ---------- ---PNDDSLL ADENVDAVLV TSWGPAHESS VLKAIKA-QK YVFCEKPLAT TAEGCMRI-- 109  ---------- ---------- ---NDYHDLI NDKDVEVVII TASNEAHADV AVAALNA-NK YVFCEKPLAV TAADCQRV-- 109  ---------- ---------- ---EDFDDMV QHADIDAVFI VSPSGFHLQQ IESALNA-GK HVFSEKPIGL DIEAIEHT-- 111  ---------- ---------- ---TDFDEMI KNKELDAIFI SSPSGFHCSQ IEKALKA-GF HVFSEKPLGL HLEETKKL-- 113  ---------- ---------- ---IDAIEA- -AADIDAVVI CTPTDTHADL IERFARA-GK AIFCEKPIDL DAERVRAC-- 105  ---------- ---------- ---KDPHELI EDPNVDAVLV CSSTNTHSEL VIACAKA-KK HVFCEKPLSL NLADVDRM-- 105  ---------- ---------- ---KNYQELL QRDDIEAVFI FTSTDMHEEI VTAAAQA-GK HIFCEKPLSM NEDEQASMAV 110  ---------- ---------- ---RDYMDIL NNPDIDTVFI FTSTDMHEEM VTAAANA-GK NIFCEKPLSM SPDEQASLNV 113  Motif II  α7  α5  α6  β7  β6  YETCRKNGVS --LHCGWMRRRDPGYQSIKE YLVSHNV--- SIQRAEFHS- FDWPLVPPE- --FLK----- TLGNIFTDLM 172  YALCRAAGAQ --LFCGWMRRHDKHFCKLAS EVQQVRASGD EIVYLRLVS- KDWPKVDPK- --FLK----- TLGSIFEDLM 190  YAMCKARGVE --LLCGWMRRSDQGYSALYK TVLTKPV--- DVCALHLVS- NDFPHMAPA- --FLK----- TLGSIFQDLM 184  YKMCDSKGVS --LYCGWTRRFDPAFVQLFK NSGN------ KIKELELIN- KDYFTPTPEA VAFLK----- TLGSIFQDFV 225  QEEVDRTGLI --LQVGFNRRFDNHFRRGRE ILSSGKVG-- RPHTIRITS- RD-PTFNLE- --YLREAA-- KAGGIYFDFV 273  RAAVQKSNVI --YQVGFNRRFDHNWKALKQ SVTNGDLG-- APSLVKARCI VD-PTYNEA- --YIRESA-- KEGGMLVDMT 183  MHAVEKSGGL K-YQVGFNRRFDRNFARVRE AVARGELG-- EPQILRVTS- RD-PTYVMS- --YLLRSA-- TEGGMMADMT 187  VEEEIKVGKR L-VQVGFMRRYDSGYVQLKE ALDNHVIG-- EPLMIHCAH- RN-PTVGDN- --Y------- TTDMAVVDTL 174  IEAEQKNGKR M-VQIGFMRRYDKGYVQLKN IIDSGEIG-- QPLMVHGRH- YN-ASTVPE- --Y------- KTPQAIYETL 174  QQVIAQHANL K-FQLGFMRRFDDSYRYAKQ LVDQGKIG-- DITLIRSYS- ID-PAAGMA- -SFVKFATSA NSGGLFLDMS 184  AKVVNGYKDK QIFMLGFMRRYDKSYLYAKE KIEDGEIG-- KPMLIRCYG- LD-PSCSIE- -SFLNFAKNN YSGGLFLDMA 187  LKVVSDTKAK --LMVGFNRRFDPHFMAVRK AIDDGRIG-- EVEMVTITS- RD-PSAPPV- -DYIK----- RSGGIFRDMT 172  IEETKKADVI --LFTGFNRRFDRNFKKLKE AVENGTIG-- KPHVLRITS- RD-PAPPPL- -DYIR----- VSGGIFLDMT 172  LRKVKEKGVT --LQVAFNRRFDPQFHEVFE LVRSGKIG-- RPQMIKITS- RD-PDLLPH- -DLIK----- RIGGLIFDFT 177  LKAVRDNNVK --LQIGFNRRSDPQFDSIHQ QVVSGTIG-- TPQVVKITS- RD-PEVTPH- -NIIK-----RIGGLLFDFT 180  Motif IV  Motif III  β11  β8  β9  β10  α8  CHDFNLIMYY MN--NT--LP KYVTAVGIDG ------GAGV WDSATASLEY PNNVVLTIIA TRNGNKVYDN SLTVLTSDSH 242  CHDFSLVDLF LE--GR--MP CTVEAGGSDT T-----GADI WDIGWARLQY PGGLSVLLES YRFGDGKYDN TATVVTRSGR 261  CHDFNLACMF MK--EE--MP VSIEAHGKDS T-----GVGI FTSATCILQY TGGRFVYMEA TRYGDGKYEN YARVYNNTGN 255  CHDLNAACLF MG---E--LP LAVEATPIDS M-----GVGI WDKVSCVLSY SENRLVKIEA DRFG--PYQQ SAKALLNDGT 293  IHDFDMLHYL ISGFQESTTV SDVVAMGSTL LAPELTELGD VDTSVVTLKL NNGCLVIIDN SRQAVYGYDQ RIEIFGADGC 353  IHDFDMCNYL LS--STAGLP VSVYTCGGSS VSPLFKEVGD VSEAVVMIRY QSGATAIIDN SRETVYGYDQ RVEIFGSKGC 261  IHDFDMLNYV IG--EHRARC TEVYALGACL IEPAVKKAGD VDTAIVTLRF ADGALASIDN SRKAVYGYDQ RVEVFGTAGC 265  VHEIDVLHWL VN--DDYESV QVIYPKKSKN ALPHLK---- -DPQIVVIET KGGIVINAEI YVNCKYGYDI QCEIVGEDGI 247  IHEIDVMHWL LN--EDYKTV KVYFPRQS-S LVTTLR---- -DPQLVVMET TSGINIVVEV FVNCQYGYDI HCDVTGEKGM 246  IHDIDVIRWF TG--KE---I DKVWAIGLNR AYPVLDKAGE LETGAALMQL EDKTMAILVA GRNAAHGYHV ETEIIGTKGM 259  IHDLDLARWY LN--SE---A EKVWAIGDAY EYEDFKEIND AETGAALVKF KNGTMGIFVA GRNCAHGYHI ETEIIGTKGT 262  IHDFDMARFL LG--EE---P VSVTATAAVL IDKAIGDAGD YDSVSVILQT ASGKQAIISN SRRATYGYDQ RIEVHGSKGA 247  IHDFDMARYI MG--EE---V EEVFADGSVL VDEEIGKAGD VDTAVVVLRF KSGALGVIDN SRRAVYGYDQ RIEVFGSKGR 247  MHDFDMARFM MQ--DE---V SEVYVKGNTL IDPSLKNIDD VDTLAVMLTF RNGGYALIDN SRRAVYGYDQ RVEVFGSEGM 252  MHDFDMARFM MG--SD---I TEVYAQAGRL VDPGLAELND YDTAIINLKF ANGAFGLIDN SRRAVYGYDQ RVEVFGSEGM 255  Motif V  α11  α9  β14  α10  β13  ILECGKE--- ---------- -----PENLT ETFMKRHEKN FKKELPFFAN IIRNNGTSG- DV--LSCVNT SILIQAATRS 301  VLRSDVVEA- ---------- ---------- MTFMERYHEA FTAEVLWYGR ALRGEAPPR- RVSPRTCEMV ARLVDLTEQA 319  TLETGKEEV- ---------- ---------H HTFMDRYLAA FSSEVEYFSR VVRGENIPR- KSTERSCVAT AVLVDLAEKS 314  KLNSLNIPG- ---------- ---------- HNVFERYDQA YSAEIEFFHK ILKGEHPDN- LCSSEHCLAT AILIEMAEKS 351  IMINNEQCSS AVVLS----- RDPPSSDKLK WFFADRYADA FLNEMEEFIN CVRNQGKP-- SVGLKDAYNA VHLATLAKQS 426  AIGENDLRSS IRLYS----- SEATKLDKIH FWFLERYELE FDSY------ ---------- ---------- ---------- 300  VVAENEAATT CRFLSEGAAG SRREQREAIP WWFTERYQDA FVAELAGFAR CVRAGPDVRP LVTCADQLRA TLLANAARES 345  IKLPEPSSIS LR-------- KEGRFSTDIL MDWQRRFVAA YDVEIQDFID SIQKKGEVS- GPTAWDGYIA AVTTDACVKA 318  AELPTVASAA VR-------- KAAKYSTDIL VDWKQRFIDA YDIEFQDFFD RLNAGLPPA- GPTSWDGYLA AVTADACVKS 317  LRIAQVPEKN LVTVM----- NEEGIIRPTS QNFPERFAQA FLSEEQAFVN SILNNQDV-- GITAEDGLQG TKAALALQEA 332  LRIGTIPEKN MVTVF----- NDKGAIRECS QGFLERFEQA YLSETEDFIN CIMEKRQP-- KITVEDGVNS TALAYACKES 335  VAAENQRPVS IEIAT----- GDGYTRPPLH DFFMTRYTEA YANEIESFIA AIEKGAEI-- APSGNDGLAA LALADAAVRS 320  IFADNVRETT VVLTD----- EQGDRGSRYL YFFLERYRDS YLEELKTFIK NVKSGEPP-- AVSGEDGKMA LLLGYAAKKS 320  AYADNVSEST VKVFN----- SQHCIMKNPL PDFTVRYREA YRTEILHFID SVLHHTPV-- VCTGEDALLA QRIAIAAQQS 325  LKAENVSGST VELYN----- ADNEIKKNPK PAFQQRYKPA YIAEMRKFVD SILNDAPL-- AATGKDVIMA QRAANAAQKS 328  Motif VI  α11  β15  ATLGGKRIPL VPKL------ ---------- ---------- ---------- ---------- ---------- ---------- 313  AKVG-HPVNA ESGAPAAKL- ---------- ---------- ---------- ---------- ---------- ---------- 334  ATSG-KRVFL TAKHRNGLSKL--------- ---------- ---------- ---------- ---------- ---------- 331  ALLR-KPLVI PPELRNSMLL QVTPVVKLPM AMMQIGFGEF GKYIQQTVMP YVSEHIRTLA IINTKNLDTI (421...483) 430  LTEH-RIVSN ITGH------ ---------- ---------- ---------- ---------- ---------- ---------- 439  ---------- ---------- ---------- ---------- ---------- ---------- ---------- ---------- 300  LRTG-RPVAP EPF------- ---------- ---------- ---------- ---------- ---------- ---------- 357  QESG-QKEKV ELKEKPEFYQSFTTVQN--- ---------- ---------- ---------- ---------- ---------- 335  QETG-NTEIV ELPSKPDFYK ---------- ---------- ---------- ---------- ---------- ---------- 334  FEKN-DIVQV ASVDKKVGA- ---------- ---------- ---------- ---------- ---------- ---------- 349  FETG-KLIEL K--------- ---------- ---------- ---------- ---------- ---------- ---------- 345  VAEK-RQISI A--------- ---------- ---------- ---------- ---------- ---------- ---------- 330  LEEK-RSVKL EEVIG----- ---------- ---------- ---------- ---------- ---------- ---------- 334  LKSG-LPVKI TSDIYL---- ---------- ---------- ---------- ---------- ---------- ---------- 340  IDTG-LPQKV DTNFQL---- ---------- ---------- ---------- ---------- ---------- ---------- 343 |
| --- | --- |

Figure S3. Protein sequence alignment of inositol dehydrogenase (IDH).

*B. subtilis* (1) (P26935); *S. typhimurium* (1) (Q8ZK57); *L.*casei (2) (A5YBJ8); *C.* tetani (2) (Q898E5); *R.* meliloti (3) (O68965); *T.* maritime (3) (Q9WYP5); *E.*coli (4) (B7MQB4); *L. antri* (4)(C8P8P1); MbIDH1 (*M. balamuthi*, m51a1_ g7332); MbIDH2 (*M. balamuthi*, m51a1_ g5305); PsIDH1 (*P. schiedti*, Pelo3967); PsIDH2 (*P. schiedti*, Pelo3265); PsIDH3 (*P. schiedti*, Pelo11053); PsIDH4 (*P. schiedti*, Pelo13800); *E. histolytica* (EHI_125740). IDHs with predicted PTS1 are marked in green. Numbers in brackets indicate IDH subgroup as described [1]. Six sequence motifs are indicated with bars. Motif I and II define NAD(H)-binding motifs while motif III-VI forms substrate-binding pocket. Residues that are identical in defined motifs are indicated with stars. Highly conserved residues that are absent in peroxisomal othologs are highlighted in yellow. Unique residues conserved in amoebae are highlighted in turquoise. The residues of putative catalytic triad involved in the catalytic mechanism of BsIDH are indicated with black triangles. GxGx_2_G NAD(H)-binding site in motif I, CEKP consensus sequence in motif II (part of a conserved motif found in sugar dehydrogenases) and GFxRRxD consensus sequence in motif III are highlighted in red [2][3][4]. Motif III of EhIDH, MbIDH1, PsIDH1, and PsIDH2 harbours CGWxRRxD.

The residues of catalytic sequence motif (helices *α*7 and *α*8) differ between subgroup 1 and the other subgroups. The active-site consensus sequence motif of subgroup 1 is Yx7(D/E)(T/S)x(I/V)HExD [1] while in subgroup 2-4 the motif is (S/I)GGxFxD(M/F)x(I/M/V)HDxD that is more similar to the active-site consensus sequence motif GGX3DX3(Y/H) found in dimeric dihydrodiol dehydrogenase, AFR, GFOR, IDHA and rhizopine catabolic protein MocA [5][6]. The catalytic sequence motif of EhIDH, MbIDH1, PsIDH1, and PsIDH2 is FLKTGxIFxDx2CHDx(N/S) seems to be unique and represent a new subgroup 5.

1. Van Straaten KE, Zheng H, Palmer DRJ, Sanders DAR. Structural investigation of myo-inositol dehydrogenase from Bacillus subtilis: Implications for catalytic mechanism and inositol dehydrogenase subfamily classification. Biochem J. 2010;432: 237–247. doi:10.1042/BJ20101079

2. Bottoms CA, Smith PE, Tanner JJ. A structurally conserved water molecule in Rossmann dinucleotide-binding domains. Protein Sci. 2009;11: 2125–2137. doi:10.1110/ps.0213502

3. Wiegert T, Sahm H, Sprenger GA. The substitution of a single amine acid residue (Ser-116 → Asp) alters NADP-containing glucose-fructose oxidoreductase of Zymomonas mobilis into a glucose dehydrogenase with dual coenzyme specificity. J Biol Chem. 1997;272: 13126–13133. doi:10.1074/jbc.272.20.13126

4. Kühn A, Yu S, Giffhorn F. Catabolism 1,5-anhydro-D-fructose in Sinorhizobium morelense S-30.7.5: Discovery, characterization, and overexpression of a new 1,5-anhydro-D-fructose reductase and its application in sugar analysis and rare sugar synthesis. Appl Environ Microbiol. 2006;72: 1248–1257. doi:10.1128/AEM.72.2.1248-1257.2006

5. Galbraith MP, Feng SF, Borneman J, Triplett EW, De Bruijn FJ, Rossbach S. A functional myo-inositol catabolism pathway is essential for rhizopine utilization by Sinorhizobium meliloti. Microbiology. 1998;144: 2915–2924. doi:10.1099/00221287-144-10-2915

6. Carbone V, Endo S, Sumii R, Chung RPT, Matsunaga T, Hara A, et al. Structures of dimeric dihydrodiol dehydrogenase apoenzyme and inhibitor complex: Probing the subunit interface with site-directed mutagenesis. Proteins Struct Funct Genet. 2008;70: 176–187. doi:10.1002/prot.21566
