# Supplementary material for: Anaerobic peroxisomes in Entamoeba histolytica metabolize myo-inositol
Source: PLoS Pathog. 2021 Nov 15;17(11):e1010041. doi: 10.1371/journal.ppat.1010041 (PMC8629394; doi:10.1371/journal.ppat.1010041)
Supplement: S4 Fig — (PDF) [file ppat.1010041.s004.pdf]

Figure S4. Purification of recombinant His-tagged *myo*-IDH using Ni-NTA column under native condition and western blot analysis of *E. histolytica* cellular fractions using polyclonal antibodies raised against purified *myo*-IDH

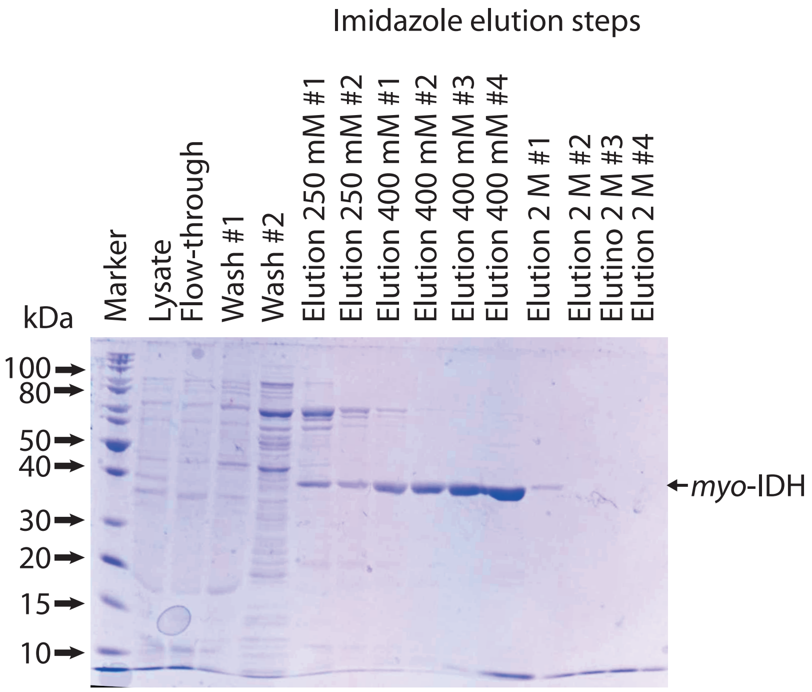

Identity of eluted IDH was confirmed by mass spectrometry and the purified IDH used for enzymology and the native molecular mass determinantion.

Western blot analysis of *E. histolytica* cellular fractions

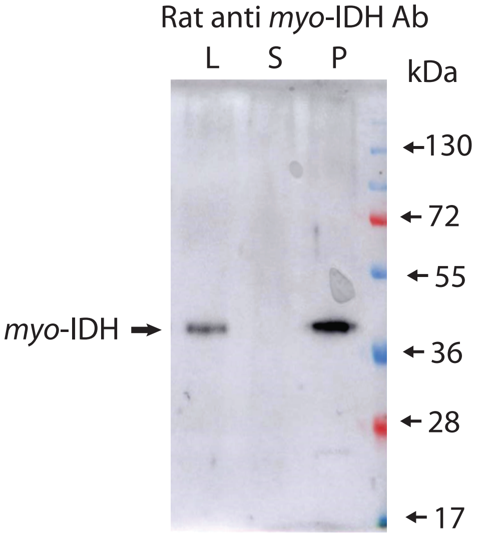

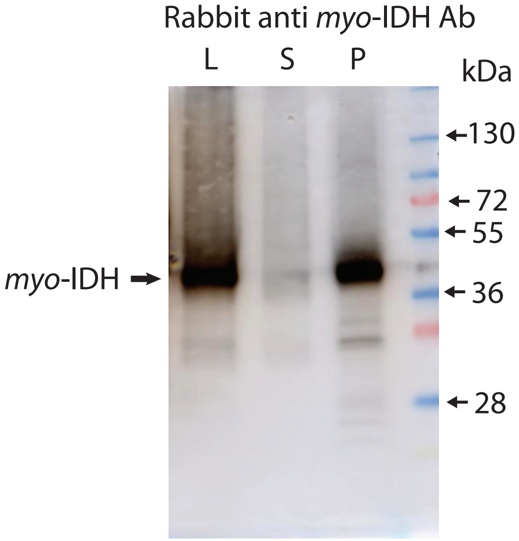

L, *E. histolytica* lysate
S, *E. histolytica* soluble (cytosolic) fraction
P, *E. histolytica* organellar fraction

Rabbit and rat polyclonal antibodies were raised against *myo*-IDH purified under denaturating conditions.
