# Supplementary material for: Anaerobic peroxisomes in Entamoeba histolytica metabolize myo-inositol
Source: PLoS Pathog. 2021 Nov 15;17(11):e1010041. doi: 10.1371/journal.ppat.1010041 (PMC8629394; doi:10.1371/journal.ppat.1010041)

Figure S5. Lineweaver–Burk plots constructed for determination of kinetic parameters of *E. histolytica* *myo*-IDH (Table 1). Each point was calculated from three measurements, error bars represent standard deviations.

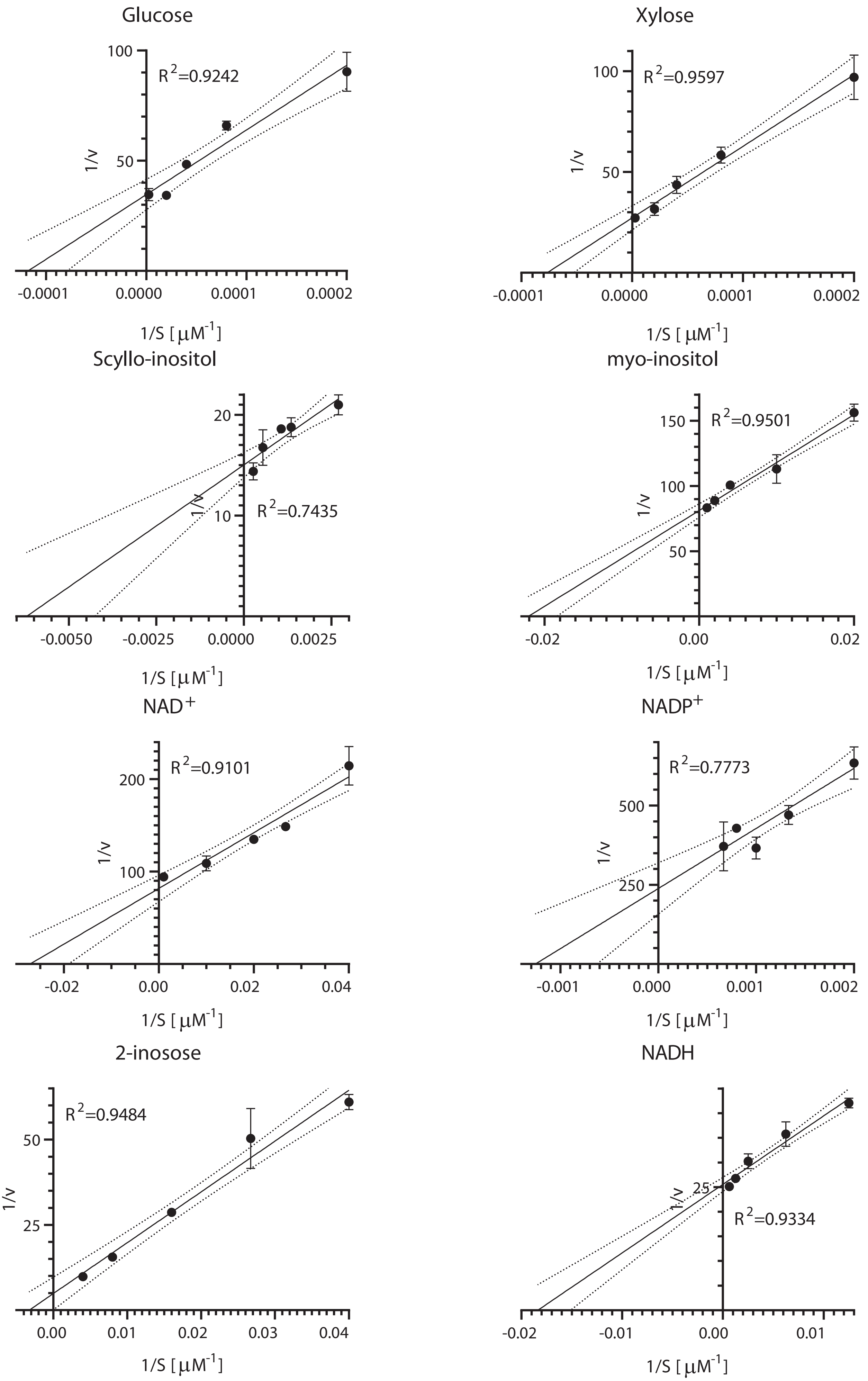

Supplement: S5 Fig — Each point was calculated from three measurements, error bars represent standard deviations. (PDF) [file ppat.1010041.s005.pdf]
