# Supplementary material for: Anaerobic peroxisomes in Entamoeba histolytica metabolize myo-inositol
Source: PLoS Pathog. 2021 Nov 15;17(11):e1010041. doi: 10.1371/journal.ppat.1010041 (PMC8629394; doi:10.1371/journal.ppat.1010041)
Supplement: S6 Fig — Superdex 200 Increase 10/300 GL column connected to BioLogic DuoFlow system (BioRad) was used for the analysis. (PDF) [file ppat.1010041.s006.pdf]

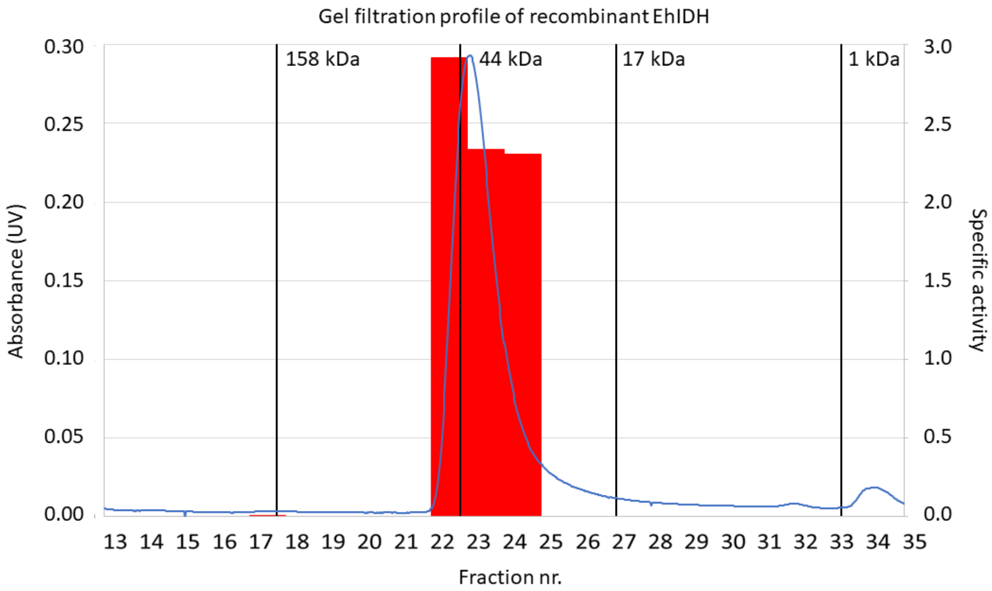

Figure S6. Native molecular mass of recombinant, His-tagged *myo*-inositol dehydrogenase estimated by size-exclusion chromatography on Superdex 200 Increase 10/300 GL column. Positions of standards in the elution profile are marked by black lines. Peak of active enzyme was recovered at the elution volume corresponding to 41 kDa. Specific *myo*-IDH activity is indicated by red columns.
