# Supplementary material for: Anaerobic peroxisomes in Entamoeba histolytica metabolize myo-inositol
Source: PLoS Pathog. 2021 Nov 15;17(11):e1010041. doi: 10.1371/journal.ppat.1010041 (PMC8629394; doi:10.1371/journal.ppat.1010041)

Figure S7. MALS analysis of *myo*-IDH. The protein sample showing the molar mass (axis on theleft) along with elution profile (axis not shown) at free different concentrations, 2.0 mg/ ml (red), 1.0 mg/ml (magenta) and 0.5 mg/ml (green).

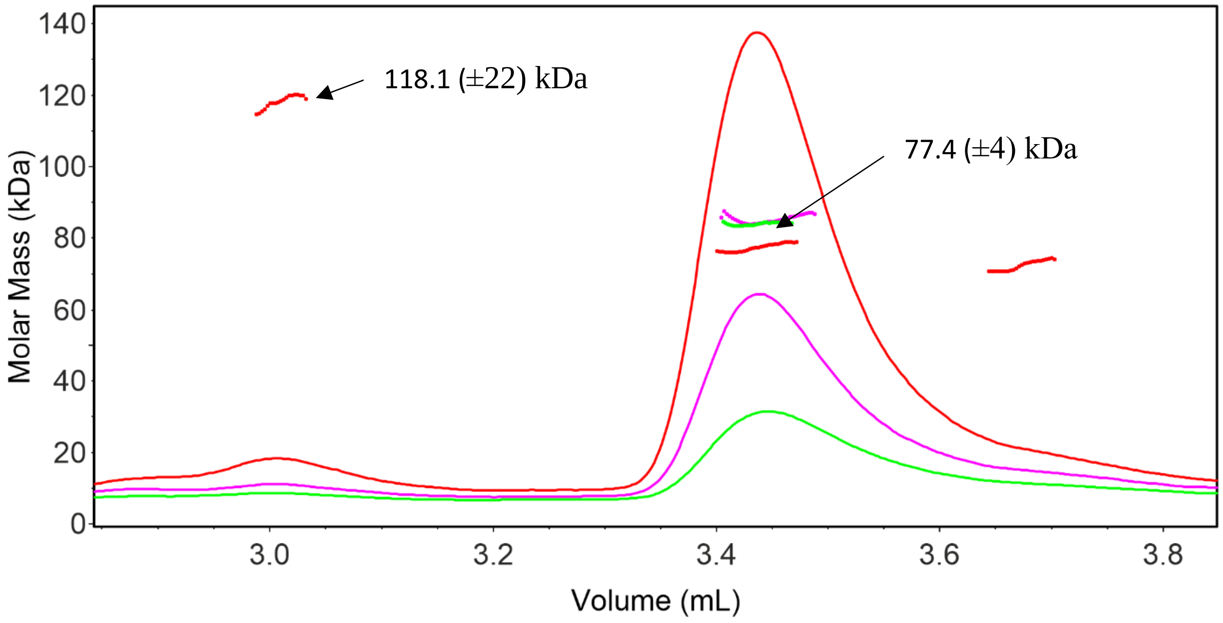

Supplement: S7 Fig — Elution profile at three different concentrations, 2.0 mg/ml (red), 1.0 mg/ml (magenta) and 0.5 mg/ml (green), with the indicated molar mass. (PDF) [file ppat.1010041.s007.pdf]
