# Supplementary figures and images for: Anaerobic peroxisomes in Entamoeba histolytica metabolize myo-inositol
Source: PLoS Pathog. 2021 Nov 15;17(11):e1010041. doi: 10.1371/journal.ppat.1010041 (PMC8629394; doi:10.1371/journal.ppat.1010041)

Figure S8. Phylogenetic tree of IDHs including their accession numbers.

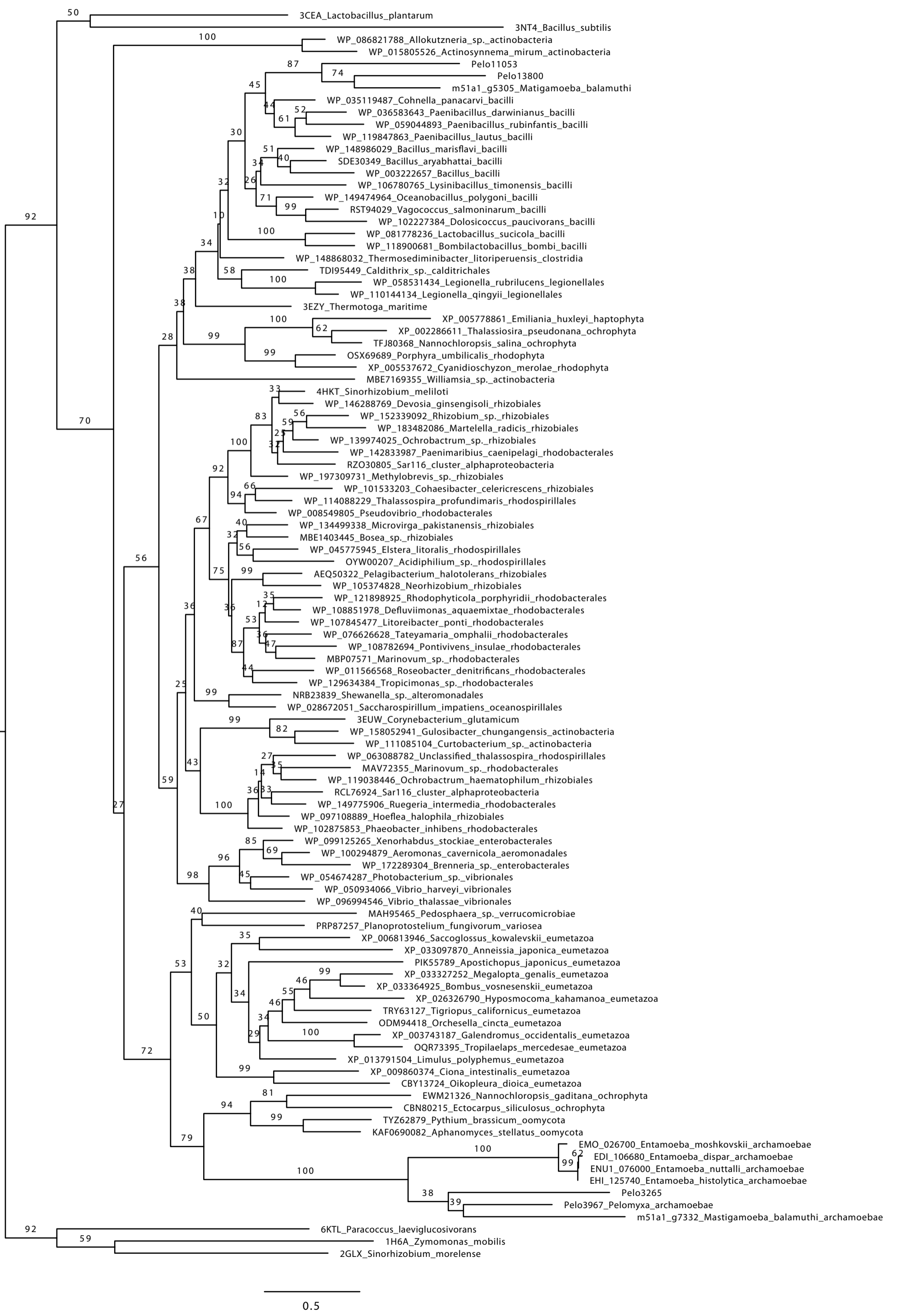

Supplement: S8 Fig — (PDF) [file ppat.1010041.s008.pdf]
