# Supplementary material for: Anaerobic peroxisomes in Entamoeba histolytica metabolize myo-inositol
Source: PLoS Pathog. 2021 Nov 15;17(11):e1010041. doi: 10.1371/journal.ppat.1010041 (PMC8629394; doi:10.1371/journal.ppat.1010041)
Supplement: S3 Table — (DOCX) [file ppat.1010041.s011.docx]

Table S3. Relative expression of PEXs and three housekeeping enzymes in *E. histolytica* determined by qRT PCR.

| Gene ID | Name | ΔCq* | baseMean** |
| --- | --- | --- | --- |
| EHI_179030 | Pex5 | 9.2 | 105.88 |
| EHI_103470 | Pex11 | 6.0 | 113.34 |
| EHI_194840 | Pex14 | 6.9 | 16.39 |
| EHI_024620 | Pex16 | 11.8 | 50.72 |
| EHI_198710 | Pex19 | 16.1 | 30.44 |
| EHI_098420 | Pyruvate kinase | 3.9 | 603.14 |
| EHI_103590 | Phosphofructokinase | 5.9 | 958.00 |
| EHI_088020 | Alcohol dehydrogenase | 3.1 | 2516.79 |

**E. histolytica* actin was used for normalization

** For comparison, mean transcriptomic data of *E. histolytica* A1np and B2p clones reported by Meyer et al., 2016 [1] are included.

**Reference**

1. Meyer M, Fehling H, Matthiesen J, Lorenzen S, Schuldt K, Bernin H, et al. Overexpression of differentially expressed genes identified in non-pathogenic and pathogenic *Entamoeba histolytica* clones allow identification of new pathogenicity factors involved in amoebic liver abscess formation. PLOS Pathog. 2016;12: e1005853. doi:10.1371/journal.ppat.1005853
