# Supplementary material for: Anaerobic peroxisomes in Entamoeba histolytica metabolize myo-inositol
Source: PLoS Pathog. 2021 Nov 15;17(11):e1010041. doi: 10.1371/journal.ppat.1010041 (PMC8629394; doi:10.1371/journal.ppat.1010041)
Supplement: S4 Table — (DOCX) [file ppat.1010041.s012.docx]

Table S4. Relative expression of *PEX* genes in *E. histolytica* transformants with respective overexpressed PEX.

| Accession  Number | Overexpressed  Gene | Fold change* | | | | |
| --- | --- | --- | --- | --- | --- | --- |
|  |  | Pex5 | Pex11 | Pex14 | Pex16 | Pex19 |
| EHI_179030 | Pex5 | 580.03 | 1.09 | 1.53 | 1.29 | 0.77 |
| EHI_103470 | Pex11 | 1.50 | 30.90 | 1.20 | 0.89 | 0.68 |
| EHI_194840 | Pex14 | 1.84 | 0.91 | 259.57 | 3.35 | 0.55 |
| EHI_024620 | Pex16 | 0.75 | 1.25 | 1.26 | 162.01 | 0.70 |
| EHI_198710 | Pex19 | 0.99 | 0.87 | 1.08 | 0.81 | 182.91 |

**E. histolytica* actin was used for normalization
