# Supplementary material for: Anaerobic peroxisomes in Entamoeba histolytica metabolize myo-inositol
Source: PLoS Pathog. 2021 Nov 15;17(11):e1010041. doi: 10.1371/journal.ppat.1010041 (PMC8629394; doi:10.1371/journal.ppat.1010041)
Supplement: S5 Table — Voxel-based colocalization with automatic thresholding was performed using ImarisColoc. Corresponding 2Dhistograms are given in S1 Fig. (DOCX) [file ppat.1010041.s013.docx]

| Figure | Green  [488 nm] | Red  [594 nm] | PCC |
| --- | --- | --- | --- |
| Fig. 4 | Pex14 | APSK | -0.048 |
|  | Pex11 | APSK | 0.241 |
|  | Pex16 | APSK | -0.244 |
|  | Pex19 | APSK | 0.023 |
|  | Pex5 | APSK | -0.072 |
| Fig. 5 | Pex14 | Atg8 | 0.033 |
|  | Pex14 | Bip1 | 0.021 |
| Fig. 9 | myoIDH | APSK | -0.151 |
|  | Pex14 | *myo*IDH | 0.512 |
|  | Pex16 | *myo*IDH | 0.260 |
|  | Pex11 | *myo*IDH | 0.547 |

Table S5. Pearson correlation coefficient (PCC) calculated for images in Fig 4, Fig 5, and Fig 9 obtained by confocal microscopy.
